# Homozygous Missense Variants in *NTNG2,* Encoding a Presynaptic Netrin-G2 Adhesion Protein, Lead to a Distinct Neurodevelopmental Disorder

**DOI:** 10.1016/j.ajhg.2019.09.025

**Published:** 2019-10-24

**Authors:** Caroline M. Dias, Jaya Punetha, Céline Zheng, Neda Mazaheri, Abolfazl Rad, Stephanie Efthymiou, Andrea Petersen, Mohammadreza Dehghani, Davut Pehlivan, Jennifer N. Partlow, Jennifer E. Posey, Vincenzo Salpietro, Alper Gezdirici, Reza Azizi Malamiri, Nihal M. Al Menabawy, Laila A. Selim, Mohammad Yahya Vahidi Mehrjardi, Selina Banu, Daniel L. Polla, Edward Yang, Jamileh Rezazadeh Varaghchi, Tadahiro Mitani, Ellen van Beusekom, Maryam Najafi, Alireza Sedaghat, Jennifer Keller-Ramey, Leslie Durham, Zeynep Coban-Akdemir, Ender Karaca, Valeria Orlova, Lieke L.M. Schaeken, Amir Sherafat, Shalini N. Jhangiani, Valentina Stanley, Gholamreza Shariati, Hamid Galehdari, Joseph G. Gleeson, Christopher A. Walsh, James R. Lupski, Elena Seiradake, Henry Houlden, Hans van Bokhoven, Reza Maroofian

**Affiliations:** 1 Division of Genetics and Genomics, Boston Children’s Hospital, Harvard Medical School, Boston, MA 02115, USA; 2 Division of Developmental Medicine, Boston Children’s Hospital, Harvard Medical School, Boston, MA 02115, USA; 3 Department of Molecular and Human Genetics, Baylor College of Medicine, Houston, TX 77030, USA; 4 Department of Biochemistry, University of Oxford, South Parks Road, Oxford, OX1 3QU, UK; 5 Department of Genetics, Faculty of Science, Shahid Chamran University of Ahvaz, Ahvaz, 6135783151, Iran; 6 Narges Medical Genetics and Prenatal Diagnosis Laboratory, Kianpars, Ahvaz, 6155689467, Iran; 7 Cellular and Molecular Research Center, Sabzevar University of Medical Sciences, Sabzevar, 009851, Iran; 8 Department of Neuromuscular Disorders, Queen Square Institute of Neurology, University College London, WC1N 3BG, London, UK; 9 Randall Children’s Hospital at Legacy Emanuel, Portland, OR 97227, USA; 10 Medical Genetics Research Centre, Shahid Sadoughi University of Medical Sciences, Yazd, Iran; 11 Section of Pediatric Neurology and Developmental Neuroscience, Department of Pediatrics, Baylor College of Medicine, Houston, TX 77030, USA; 12 Howard Hughes Medical Institute, Boston Children’s Hospital, Boston, MA 02115, USA; 13 Department of Medical Genetics, Kanuni Sultan Suleyman Training and Research Hospital, Istanbul, 34303, Turkey; 14 Department of Paediatric Neurology, Golestan Medical, Educational, and Research Center, Ahvaz Jundishapur University of Medical Sciences, Ahvaz 6163764648, Iran; 15 Pediatric Neurology and Metabolic Division, Cairo University Children Hospital, Egypt; 16 Department of Pediatric Neurology, ICH and SSF Hospital Mirpur, Dhaka, 1216, Bangladesh; 17 Department of Human Genetics, Donders Institute for Brain, Cognition and Behaviour, Radboud University Medical Center, 6500 HB, Nijmegen, the Netherlands; 18 CAPES Foundation, Ministry of Education of Brazil, 549 Brasília, Brazil; 19 Department of Radiology, Boston Children’s Hospital, Boston, MA 02115, USA; 20 Hasti Genetic Counseling Center of Welfare Organization of Southern Khorasan, Birjand, Iran; 21 Genome Research Division, Human Genetics Department, Radboud University Medical Center, 6500 HB, Nijmegen, the Netherlands; 22 Health Research Institute, Diabetes Research Center, Ahvaz Jundishapur University of Medical Sciences, Ahvaz, Iran; 23 GeneDx, Gaithersburg, MD 20877, USA; 24 Department of Genetics, University of Alabama at Birmingham, Birmingham, AL 35294, USA; 25 Department of Neurology, Faculty of Medicine, Bam University of Medical Sciences, Bam, Iran; 26 Human Genome Sequencing Center, Baylor College of Medicine, Houston, TX 77030, USA; 27 Laboratory for Pediatric Brain Disease, Howard Hughes Medical Institute, Department of Neurosciences, University of California, San Diego, La Jolla, CA 92093, USA; 28 Department of Medical Genetics, Faculty of Medicine, Ahvaz Jundishapur University of Medical Sciences, Ahvaz 6135715794, Iran; 29 Department of Pediatrics, Baylor College of Medicine, Houston, TX 77030, USA; 30 Texas Children’s Hospital, Houston, TX 77030, USA

**Keywords:** developmental delay, intellectual disability, autism, neurodevelopmental disorder, *NTNG2*

## Abstract

*NTNG2* encodes netrin-G2, a membrane-anchored protein implicated in the molecular organization of neuronal circuitry and synaptic organization and diversification in vertebrates. In this study, through a combination of exome sequencing and autozygosity mapping, we have identified 16 individuals (from seven unrelated families) with ultra-rare homozygous missense variants in *NTNG2*; these individuals present with shared features of a neurodevelopmental disorder consisting of global developmental delay, severe to profound intellectual disability, muscle weakness and abnormal tone, autistic features, behavioral abnormalities, and variable dysmorphisms. The variants disrupt highly conserved residues across the protein. Functional experiments, including *in silico* analysis of the protein structure, *in vitro* assessment of cell surface expression, and *in vitro* knockdown, revealed potential mechanisms of pathogenicity of the variants, including loss of protein function and decreased neurite outgrowth. Our data indicate that appropriate expression of *NTNG2* plays an important role in neurotypical development.

## Main Text

Based on studies in invertebrates and chicken and mouse, netrins are considered to be the paradigmatic axon guidance molecules, yet the essential role of this family of proteins in humans remains unclear. Members of the classical netrin family are secreted proteins that include UNC6 (uncoordinated-6) in *C. elegans* and netrins NTN1–4 in vertebrates.^1^, ^2^ Netrin-G proteins (NTNG1 and NTNG2) are distinct from classical netrins in that they are vertebrate-specific, membrane-bound proteins tethered to the plasma membrane by glycosyl phosphatidylinositol (GPI) anchors.^3^ *NTNG1* (MIM: 608818) and *NTNG2* are predominantly expressed in a non-overlapping and complementary pattern in specific neuronal subsets of the developing and mature central nervous system.^4^, ^5^, ^6^ The proteins interact with the extracellular region of their specific netrin-G ligand receptors NGL-1/LRRC4C (MIM: 608817) and NGL-2/LRRC4, respectively.^7^ Selectivity in binding between netrin-G molecules and their cognate receptors is mediated by the interactions of three loops of the laminin domain, and the extracellular leucine rich repeats (LRR) domain of NGLs results in a molecular hand-clasp interaction of high affinity.^8^

*NTNG2* encodes netrin-G2, a vertebrate-specific protein that is part of a distinct functional sub-class of the highly conserved netrin family. The netrin family provides axonal guidance cues during central nervous system development.^9^ *NTNG2* is located on 9q34.13, and the canonical transcript consists of eight exons including seven coding exons; it encodes a 530-amino-acid protein. *NTNG2* demonstrates evidence of missense constraint in the ExAC database, with a Z score of 4.34, and review of population-based (gnomAD) and ethnically diverse in-house databases reveals an absence of homozygous damaging and/or deleterious variants. Despite this constraint, its potential role in human genetic disease is not clear. Here we show that bi-allelic missense variants in *NTNG2* cause a distinctive neurological and behavioral disorder that highlights the importance of this family of genes in human nervous system development.

**Figure 1:**
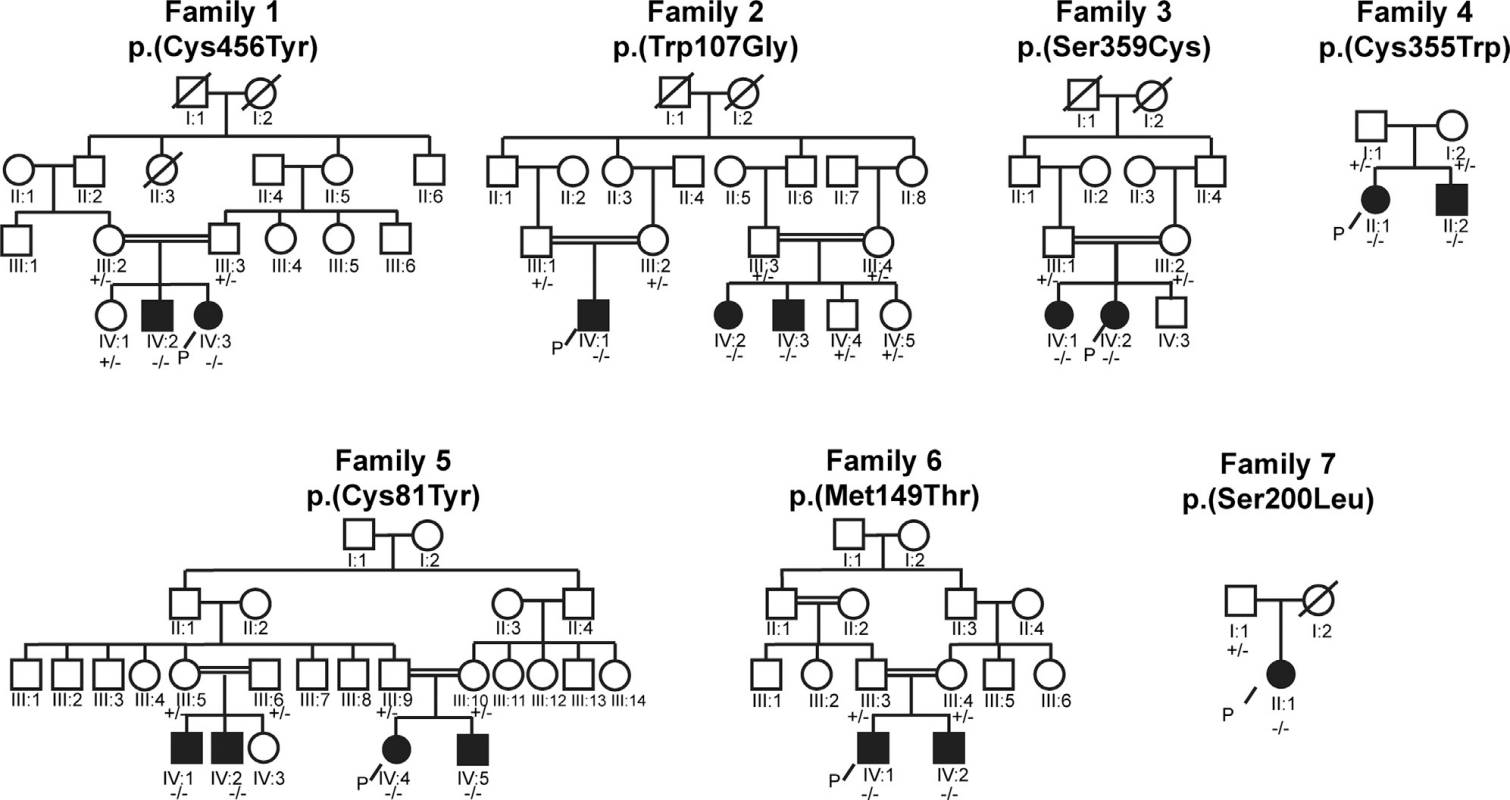
Pedigrees of All Families with Affected Individuals and Variants and Segregation Findings + indicates wild-type allele, - indicates variant allele, P indicates proband.

We have identified 16 individuals (from seven unrelated families) who have ultra-rare bi-allelic variants in *NTNG2* and who present with shared clinical features of a neurodevelopmental disorder. Consent for clinical data and biological material collection, use, and storage was obtained from all participating families after written informed consent was provided, and studies for each family were approved by their respective institutional review boards (see Supplemental Data for further details). Following genomic DNA extraction from blood, exome sequencing, and homozygosity mapping, we identified 16 individuals in seven unrelated families from different parts of Iran (Families 1, 2, 3), Mexico (Family 4), Turkey (Family 5), Egypt (Family 6), and Bangladesh (Family 7) who have a similar clinical phenotype and have homozygous missense variants in *NTNG2* (Figure S1,Table S1). Researchers and physicians for all families were connected using the GeneMatcher/Matchmaker exchange.^10^, ^11^ All families except for Families 4 and 7 had a known history of consanguinity, and all of the variants were segregated in the affected families in accordance with Mendelian expectations for a recessive disease trait (Figure 1). Autozygosity mapping for Families 4 and 7 revealed distant relatedness, and parents of the proband in Family 7 come from the same village (Figure S2, Table S2). There was no evidence of neuropsychiatric disorders in the heterozygous family members presented here.

**Figure 2:**
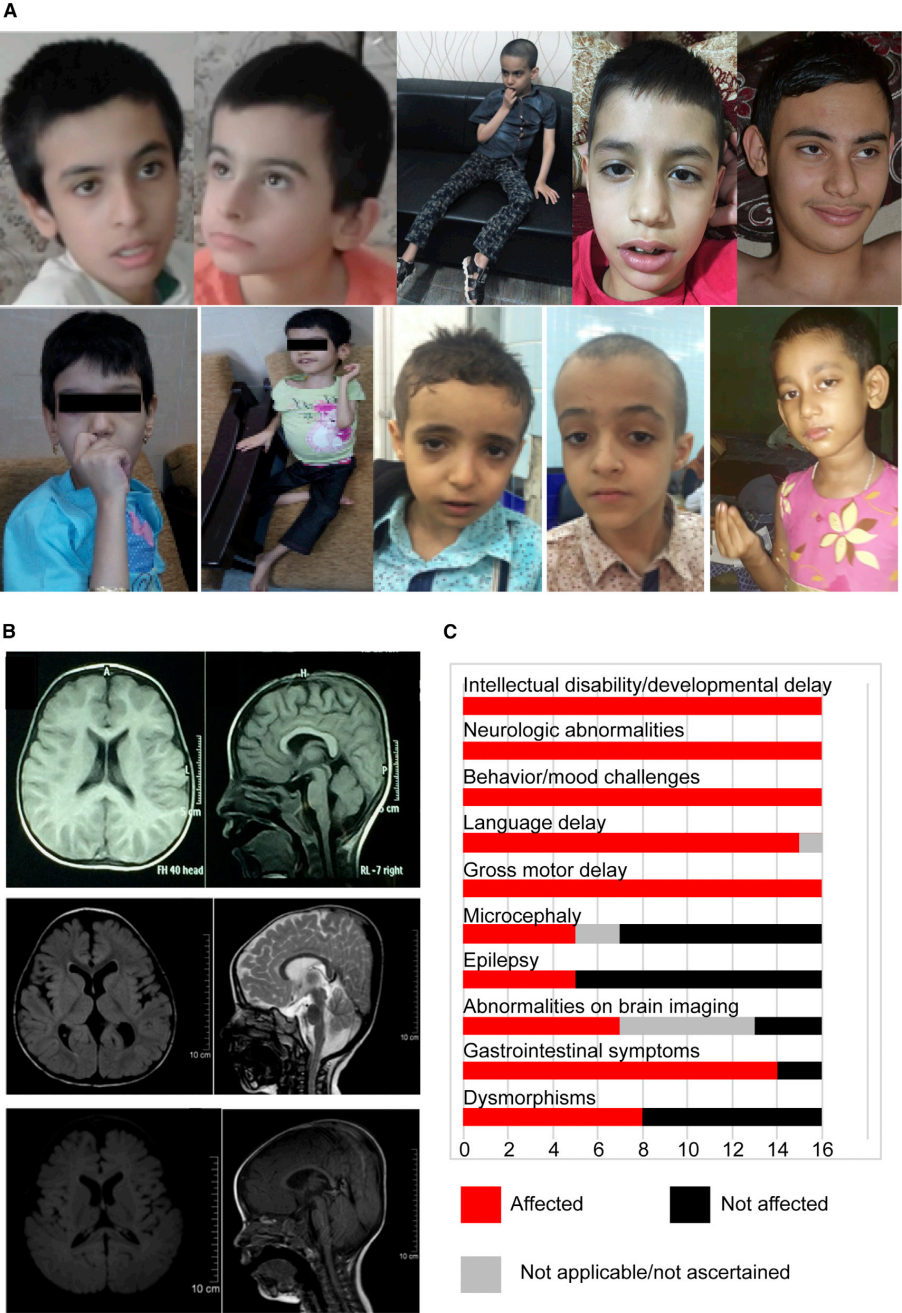
Clinical Features of Affected Individuals (A) Representative photographs demonstrating clinical features of affected individuals; these features include facial features, muscular atrophy, and hand stereotypy. Top row from left to right: Family 2 IV:2, IV:3, IV:1; Family 1 IV:3, IV:2. Bottom row: Family 3 IV:1, IV:2; Family 6 IV:2, IV:1; Family 7 II:1. (B) Representative MRIs of affected individuals, demonstrating decreased brain volume. From top to bottom: Family 6 IV:2; Family 5 IV:4; Family 5 IV:5. (C) Bar graph summarizing proportions of various clinical findings affecting individuals.

Clinical features of affected individuals are presented in Table 1. Affected individuals presented with global developmental delay with severe to profound intellectual disability; the majority were non-verbal and non-ambulatory. Most individuals also had features of autism and all were noted to have mood and/or behavioral challenges, many of which were similar to those seen in Rett syndrome, such as hand stereotypy, episodes of laughing and/or screaming, and bruxism, and in Angelman syndrome (Videos S1–S4). Gastrointestinal symptoms, including constipation and bloating, were also common. Growth parameters were below average, and four individuals had documented failure to thrive. Secondary microcephaly was also observed. Dysmorphic features were variable and included low-set ears, hypotelorism, and frontal bossing (Figure 2A). Neurologically, hypotonia in infancy and muscle weakness and/or atrophy were common findings. Five individuals had early-onset seizures, and four were noted to have ocular findings of esotropia, nystagmus, or strabismus. Brain imaging, conducted in both infancy and childhood, demonstrated findings ranging from normal to mild brain atrophy with white matter abnormalities (Figure 2B). In summary, affected individuals display a neurodevelopmental disorder of severe-to-profound intellectual disability with marked motor involvement and mood and behavioral challenges including autistic features, as well as poor growth and facial dysmorphisms. Detailed phenotypic descriptions are provided in Table 1, Table S1, Figure 2, and the Supplemental Note: Case Reports in Supplemental Data.

**Table 1:** Clinical Features of Affected Individuals

**Family**	**1**	**1**	**2**	**2**	**2**	**3**	**3**	**4**	**4**	**5**	**5**	**5**	**5**	**6**	**6**	**7**
Individual	IV:2	IV:3	IV:1	IV:2	IV:3	IV:1	IV:2	II:1	II:2	IV:1	IV:2	IV:4	IV:5	IV:1	IV:2	II:1
Age (years)	18	10	11	16	9	15	11	11	21	11	1.25	9	5	11	8	3
Sex	M	F	M	M	F	F	F	F	M	M	M	F	M	M	M	F
ID/GDD	+	+	+	+	+	+	+	+	+	+	+	+	+	+	+	+
Motor delay	+	+	+	+	+	+	+	+	+	+	+	+	+	+	+	+
Language delay	+	+	+	+	+	+	+	+	+	+	NA	+	+	+	+	+
Autistic features/stereotypy	+	+	+	+	+	+	+	+	-	+	NA	+	+	+	-	+
Hyperactivity	-	+	+	+	+	-	-	-	+	-	-	-	-	+	+	-
Screaming/laughing spells	+	+	+	+	+	+	+	+	+	+	NA	+	-	+	-	+
Self-injury/hand-biting	+	+	+	+	+	-	-	+	+	-	-	-	-	-	-	-
Bruxism	-	+	+	+	+	-	-	-	-	-	NA	+	+	+	-	-
Hypotonia in infancy	+	+	+	+	+	+	+	+	+	+	+	+	+	-	-	+
Nonambulatory	+	+	+	+	+	+	+	+	+	+	NA	+	+	-	-	-
Brain imaging abnormalities	-	NA	+	NA	NA	-	+	-	NA	NA	NA	+	+	+	+	+
Seizures	-	-	+	-	-	-	-	+	+	-	-	-	-	+	-	+
Microcephaly	-	+	-	+	+	-	-	+	NA	NA	-	+	-	-	-	-
Secondary Microcephaly		+		NA	+			NA				NA				
Dysmorphic features	-	-	+	-	-	+	+	-	-	NA	+	+	+	+	+	-
Ophthalmologic features	-	-	-	-	-	+	+	-	-	-	-	+	+	-	-	-
GI symptoms	+	+	+	+	+	+	+	+	+	+	+	+	+	-	-	+

ID/GDD, intellectual disability/global developmental delay; GI, gastrointestinal; -, absent; +, present; NA, not ascertained/not applicable

Video S1. Affected Individuals Demonstrating Hand and Facial Stereotypy, Part 1

Video S2. Affected Individuals Demonstrating Hand and Facial Stereotypy, Part 2

Video S3. Affected Individuals Demonstrating Stereotypy and Body Rocking

Video S4. Affected Individual Demonstrating Jerky Movements Movements are similar to those observed in Angelman syndrome.

**Figure 3:**
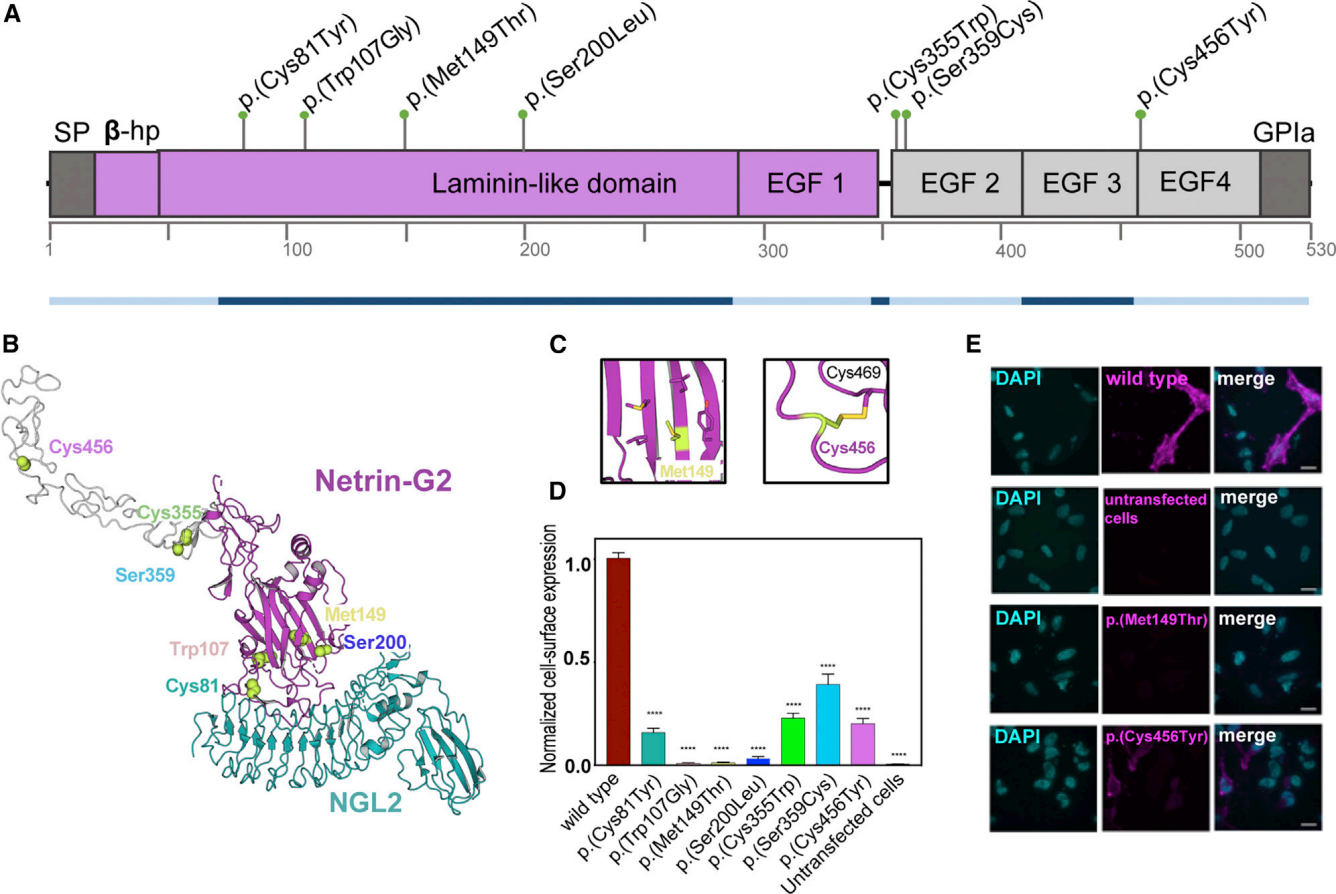
Structural Mapping and Cell Surface Expression (A) Netrin-G2 domain overview. The positions of altered residues relative to the protein domains are indicated. Domain nomenclature is: SP, signal peptide; β-hp, N-terminal β-hairpin domain; EGF, epidermal-growth factor like; GPIa, GPI anchor. Corresponding exons are represented underneath the domain organization in blue. (B) Full-length model of netrin-G2 based on the crystal structure of the Laminin-like domain and EGF1^8^ (purple) and on homology models of EGF2-4 (gray), in complex with its ligand NGL2 (cyan). The residues that are mutated in the presented variants are indicated as green spheres. (C) Close-up views of Met149 and Cys456 residues as found in the structural model shown in panel B. For close-up views of the other mutated residues, see Figure S3. (D) The quantification of the cell surface expression levels of wild type (WT) and mutant netrin-G2 constructs (see panel E) is shown (mean ± SEM). The variants show significantly reduced cell-surface expression compared to the WT (^∗∗∗∗^p < 0.0001). (E) Netrin-G2 constructs were expressed in HeLa cells with an N-terminal flag tag. Flag-tagged protein was detected via cell-surface immunostaining (magenta). DAPI (blue) highlights cell nuclei. Representative images are shown for WT netrin-G2, untransfected cells (negative control), p.Met149Thr and p.Cys456Tyr variants. Representative images of other variants are shown in Figure S3. Scale bar is 15 μm.

The ultra-rare variants we identified from family based genomic studies in the above individuals were notable for several reasons (Table S2). All variants were absent from both local ethnically diverse in-house databases, as well as large population databases. Because most of the *NTNG2* missense variants observed are rare to their specific “clan,” they may reflect variants that arose recently and according to the clan genomics hypothesis are therefore expected to have a larger influence on disease.^12^ All variants were predicted by a majority of prediction tools (FATHMM, MutationAssessor, MutationTaster, PolyPhen-2, SIFT, PROVEAN, and CADD) to be likely damaging to protein function, and genomic evolutionary rate profiling (GERP) indicated that these sites may be under evolutionary constraint (Table S2). In fact, all variants impact residues conserved in NTNG1, a result that gives further evidence for the argument that they are pathogenic. Annotation of the variant locations on the protein domains of NTNG2 revealed that they are not confined to one domain, but they fall within the laminin and EGF domains and are predicted to disrupt structural motifs within NTNG2 (Figure 3A). No other alternative candidate variants common to the families were identified (Table S3).

The available NGL2/netrin-G2 crystal structure contains a model of the netrin-G2 N terminus up to the first EGF domain. We used MODELER^13^ to create a homology for the EGF domains 2–4, which were not included in that crystal structure. Using these models, we found that the variants are located in the laminin, EGF2, or EGF4 domain (Figure 3B–3C, Figure S3). In addition to possible effects on specific protein-to-protein interaction sites, this suggests a more global mechanism of functional disruption. Strikingly, we found that four of the seven, i.e., 57% of the variants, involve the loss or addition of cysteine residues (GenBank: NM_032536.3: c.242G>A [p.Cys81Tyr], c.1065C>G [p.Cys355Trp], c.1076C>G [p.Ser359Cys], c.1367G>A [p.Cys456Tyr]). Given that the cysteine content of NTNG2 is only 7.9% in humans, the enrichment for cysteine variants in this cohort suggests a mechanism of pathogenicity. Due to the oxidizing environment in the endoplasmic reticulum (ER) and extracellular space, cysteine residues found in extracellular proteins typically appear in pairs and form disulfide bridges. Such bridges can stabilize a protein by reducing the entropy of the unfolded state and/or they can facilitate the path to the native state if they link parts of a protein that must come into contact early during a folding reaction.^14^ Unpaired exposed cysteines are detected by the ER quality control machinery and targeted for refolding or degradation.^14^ We hypothesize that the *NTNG2* variants involving cysteine could have a negative effect on protein stability and cell surface expression.

Three out of the seven variants do not involve cysteines: c.599C>T (p.Ser200Leu), c.319T>G (p.Trp107Gly), and c.446T>C, (p.Met149Thr). For both p.Trp107Gly and p.Met149Thr, a large hydrophobic residue (Trp or Met) is changed to either one lacking a side chain (Gly) or one bearing a small polar side chain (Thr). Both of these residues form part of the hydrophobic core that stabilizes the folding of the netrin-G2 laminin domain. Disruption likely causes protein misfolding and lack of expression at the cell surface. Thus the consequence of both types of variants, cysteine-dependent or hydrophobic core disruptive, is potentially a similar reduction in protein stability and expression at the cell surface. The p.Ser200Leu variant does not fit into either of the above categories, with Ser200 located at the periphery of the laminin domain. It is also located ∼0.9 nm away from the surface of NGL2 as found in the crystal structure (Figure S3). With typical hydrogen bonds about 0.25 nm in length, Ser200 is not interacting directly with NGL2, although allosteric effects could still influence the netrin-G2 binding loops.

We tested all variants for function by overexpressing wild-type (WT) and variant netrin-G2 constructs in HeLa cells and assessing their presence at the cell surface through the use of indirect immunofluorescence and immunoblotting validation (Figure S4). All variants displayed substantially decreased cell surface expression as compared to WT (Figures 3D–3E). Notably, some of these variants had more cell surface expression compared to others, suggesting that some netrin-G2 may still be localized in these individuals. The variants may nevertheless show deficient ligand-receptor binding or signaling since we did not observe a clear association with cell surface expression levels and clinical phenotype severity.

**Figure 4:**
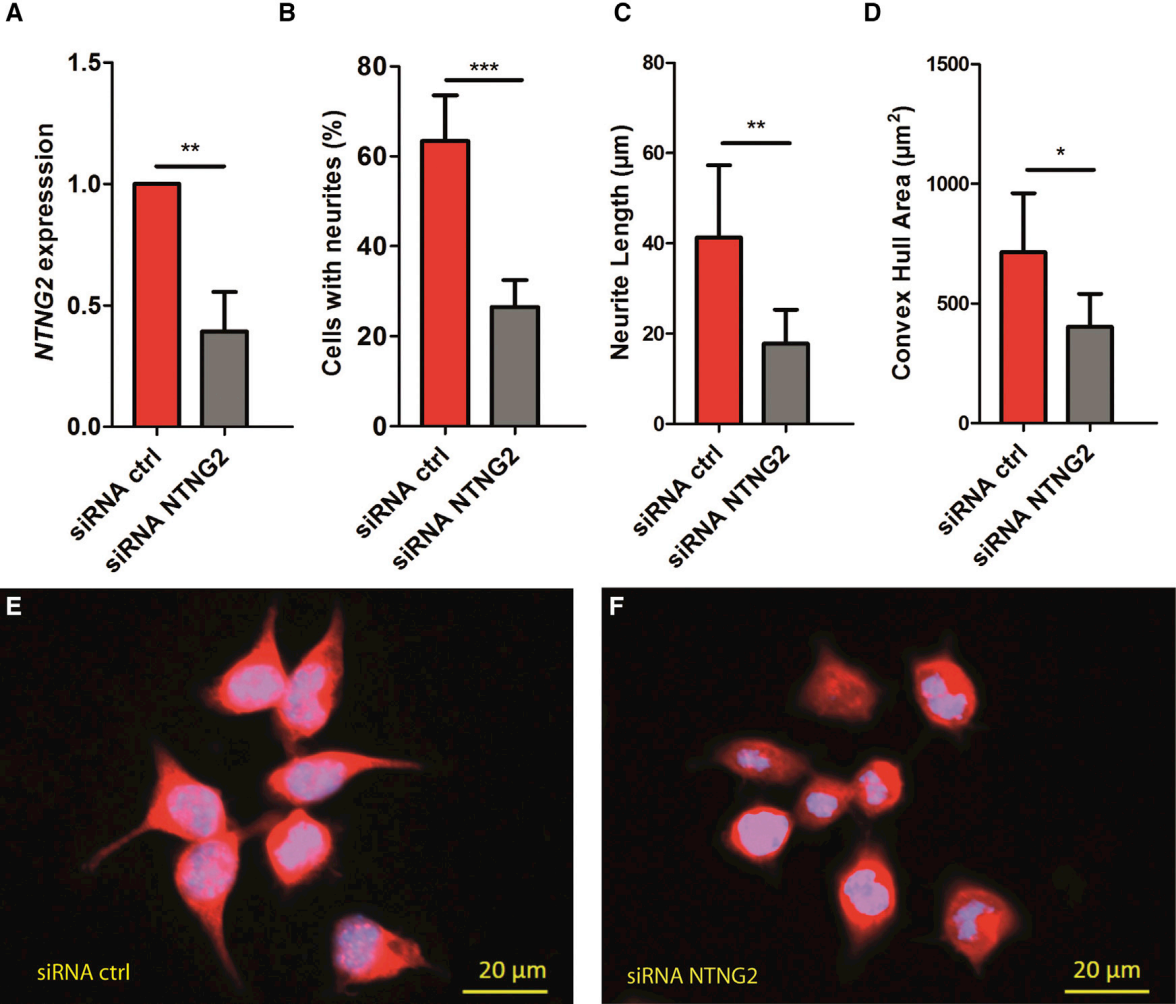
*Ntng2* Knockdown in N2a Cells (A) Knockdown of endogenous *Ntng2* by *Ntng2*-specific siRNAs as normalized to control siRNA. Results of quantitative RT-PCR 30 h post-transfection. (B–D) Effects of *Ntng2* knockdown on neurite outgrowth. Data presented as mean and SD; ^∗^p < 0.05, ^∗∗^p < 0.01, ^∗∗∗^p < 0.001. Quantification was conducted by counting the absolute number of cells with neurites (B), measuring the neurite length by NeuroLucida tracing (C), and quantification of the Convex hull area (D). All analyses show a significant reduction of neurite number and length as a consequence of *Ntng2* knockdown. (E–F) Example of the N2a appearance at 30 h post-transfection with control (E) and *Ntng2*-specific siRNA (F). Visualization was done using MAP2 counterstaining (red).

Given the decreased cell surface expression pattern observed in all seven variants, we sought to determine the more global effects of *NTNG2* loss of function. Using siRNA to target endogenous *Ntng2* expression in mouse N2A cells, we first confirmed that transfection with our *Ntng2-*specific siRNA led to decreased expression with quantitative polymerase chain reaction (Figure 4A). We next assessed neurite outgrowth and found a significant reduction for all parameters assessed, which included neurite number, neurite length, and convex hull area—a measurement used for measuring dendritic field (Figures 4B–4F). These findings demonstrate a potential mechanism by which the *NTNG2* variants may contribute to pathological neurodevelopment.

Netrin signaling has been implicated in neurologic and psychiatric disorders. For example, conditional *Ntng1* knockout in distinct neuronal subtypes is associated with alterations in fear and anxiety-like behaviors in rodent models, and abnormal expression of *NTNG*2 has been found in the human brain in refractory epilepsy.^15^, ^16^ Studies of *Ntng2* and *Ngl2* knockout mice have shown that both types of mutant mice have an identical phenotype of lack of behavioral startle in response to acoustic stimulus, with no structural abnormalities noted in the inner ear.^17^ Single-nucleotide polymorphisms (SNPs) and differential expression patterns of *NTNG1* and *NTNG*2 have been associated with schizophrenia and bipolar disorder in humans.^18^, ^19^, ^20^ A *de novo* genomic rearrangement involving *NTNG1* was proposed to potentially cause features of Rett syndrome in an isolated individual.^21^ Additionally, *de novo* missense variants in *NTNG1* were reported in two individuals with autism spectrum disorder.^22^ An *in vitro* study of variants in the histone demethylase *KDM5C* (Lysine demethylase 5C [MIM: 314690]), which is known to cause intellectual disability, showed that *NTNG2* seemed to be important in mediating effects on neurite growth and length; these results are consistent with our findings here.^23^ In fact, several clinical features, in addition to intellectual disability, are shared between these two disorders, including variable neurologic, behavioral, and dysmorphic features. Extensive behavioral battery on *Ntng2* knockout mice demonstrated marked deficits in learning, memory, and visual and motor functioning.^24^ Although *NTNG2* does not appear to be necessary for axon guidance, it has been shown to be important in the laminar distribution of its receptors and synaptic plasticity.^25^, ^26^ A homozygous founder frameshift variant in *NTNG2* has recently been identified in eight individuals from four families with a similar clinical phenotype, and this further strengthens the evidence supporting the pathogenicity of the variants presented here.^27^

Other genes involved in netrin signaling have also been implicated in neurodevelopmental disorders in isolated case reports, some of which have involved examples of *de novo* variation.^28^ Specifically, variations in *LRRC4C* and *LRRC4* have both been associated with intellectual disability and autism.^29^, ^30^ Furthermore, functional work in mice has shown that *LRRC4* expression regulates N-methyl-D-aspartate receptor (NMDAR)-dependent synaptic plasticity and prevents autistic-like behaviors.^31^ *LRRC4C* and *LRRC4* have both been shown to be important in hippocampal synapse formation and function.^32^, ^33^ The marked findings of severe intellectual disability and autistic features in our cohort are particularly intriguing given the unique role of *NTNG2* in vertebrates. As we previously mentioned, netrin-g family members express in distinct, non-overlapping, and complementary neuronal circuits, suggesting a role in establishing appropriate neuronal patterning. This neuronal compartmentalization parallels distinct behavioral compartmentalization, as in mouse knockout models, *Ntng2* knockouts demonstrated sensorimotor, spatial memory, working memory, procedural learning, and attentional deficits, while *Ntng1* knockouts demonstrated distinct learning and fear conditioning deficits.^24^ Our findings here, in conjunction with the known role of NTNG2 in the control of synaptic plasticity and postsynaptic membrane organization, illustrate the clinical relevance of these neuronal functions to higher cognitive processes. In fact, given the profound finding of intellectual disability in the individuals presented here, it is intriguing that *NTNG2* expression is enriched in the human claustrum, an enigmatic brain region posited to play a role in the integration of conscious perception.^34^

Our work provides the groundwork for establishing a genotype-to-phenotype relationship with *NTNG2* variants, and establishes an initial description of the clinical spectrum. *NTNG2* should be considered in the clinical evaluation of children with severe intellectual disability and neuropsychiatric symptoms. In addition to identification by exome sequencing, it will be important to add *NTNG2* to clinical gene-panel tests for intellectual disability given the marked yet variable clinical phenotype. In summary, our results implicate rare bi-allelic missense *NTNG2* variants in the pathobiology of a neurodevelopmental disorder consisting of severe intellectual disability, autistic features, and motor impairment. Our findings provide strong clinical and functional evidence for the importance of the appropriate expression of *NTNG2* in neurodevelopment.

## Declaration of Interests

Baylor College of Medicine (BCM) and Miraca Holdings have formed a joint venture with shared ownership and governance of Baylor Genetics (BG), which performs clinical microarray analysis and clinical exome sequencing. J.R.L. serves on the Scientific Advisory Board of BG. J.R.L. has stock ownership in 23andMe, is a paid consultant for Regeneron Pharmaceuticals, has stock options in Lasergen, and is a co-inventor on multiple United States and European patents related to molecular diagnostics for inherited neuropathies, eye diseases, and bacterial genomic fingerprinting. The other authors declare no competing interests.

## Accession Numbers

The accession numbers for the variants reported in this paper are ClinVar SCV000994967.1, SCV000994968.1, SCV000994969.1, SCV000994970.1, SCV000994971.1, SCV000994972.1, and SCV000994973.1.

LOVD variant identification numbers are 597120–597126.

## Web Resources

CADD, https://cadd.gs.washington.edu/
ExAC, http://exac.broadinstitute.org/
FATHMM web server, http://fathmm.biocompute.org.uk/
GeneMatcher, https://genematcher.org/
GERP, http://mendel.stanford.edu/sidowlab/downloads/gerp/index.html
gnomAD, https://gnomad.broadinstitute.org/
MutationAssessor web server, http://mutationassessor.org/r3/
MutationTaster, http://www.mutationtaster.org/
Online Mendelian Inheritance in Man, https://www.omim.org
PolyPhen-2, http://genetics.bwh.harvard.edu/pph2/
PROVEAN, http://provean.jcvi.org/index.php
SIFT, https://sift.bii.a-star.edu.sg/

## Supplemental Data

Document S1. Supplemental Note, Figures S1–S4, Table S1 Legend, Tables S2–S3, Supplemental Methods, Acknowledgments, Author Contributions, and Supplemental References

Document S2. Table S1

Document S3. Article plus Supplemental Data

## References

[1] Serafini T., Kennedy T.E., Galko M.J., Mirzayan C., Jessell T.M., Tessier-Lavigne M. (1994). The netrins define a family of axon outgrowth-promoting proteins homologous to C. elegans UNC-6. Cell.

[2] Dickson B.J. (2002). Molecular mechanisms of axon guidance. Science.

[3] Cirulli V., Yebra M. (2007). Netrins: beyond the brain. Nat. Rev. Mol. Cell Biol..

[4] Nakashiba T., Nishimura S., Ikeda T., Itohara S. (2002). Complementary expression and neurite outgrowth activity of netrin-G subfamily members. Mech. Dev..

[5] Meerabux J.M., Ohba H., Fukasawa M., Suto Y., Aoki-Suzuki M., Nakashiba T., Nishimura S., Itohara S., Yoshikawa T. (2005). Human netrin-G1 isoforms show evidence of differential expression. Genomics.

[6] Lin J.C., Ho W.H., Gurney A., Rosenthal A. (2003). The netrin-G1 ligand NGL-1 promotes the outgrowth of thalamocortical axons. Nat. Neurosci..

[7] Woo J., Kwon S.K., Kim E. (2009). The NGL family of leucine-rich repeat-containing synaptic adhesion molecules. Mol. Cell. Neurosci..

[8] Seiradake E., Coles C.H., Perestenko P.V., Harlos K., McIlhinney R.A., Aricescu A.R., Jones E.Y. (2011). Structural basis for cell surface patterning through NetrinG-NGL interactions. EMBO J..

[9] Nakashiba T., Ikeda T., Nishimura S., Tashiro K., Honjo T., Culotti J.G., Itohara S. (2000). Netrin-G1: a novel glycosyl phosphatidylinositol-linked mammalian netrin that is functionally divergent from classical netrins. J. Neurosci..

[10] Philippakis A.A., Azzariti D.R., Beltran S., Brookes A.J., Brownstein C.A., Brudno M., Brunner H.G., Buske O.J., Carey K., Doll C. (2015). The Matchmaker Exchange: a platform for rare disease gene discovery. Hum. Mutat..

[11] Sobreira N., Schiettecatte F., Valle D., Hamosh A. (2015). GeneMatcher: a matching tool for connecting investigators with an interest in the same gene. Hum. Mutat..

[12] Lupski J.R., Belmont J.W., Boerwinkle E., Gibbs R.A. (2011). Clan genomics and the complex architecture of human disease. Cell.

[13] Webb B., Sali A. (2016). Comparative Protein Structure Modeling Using MODELLER. Curr. Protoc. Bioinformatics..

[14] Feige M.J., Hendershot L.M. (2011). Disulfide bonds in ER protein folding and homeostasis. Curr. Opin. Cell Biol..

[15] Pan Y., Liu G., Fang M., Shen L., Wang L., Han Y., Shen D., Wang X. (2010). Abnormal expression of netrin-G2 in temporal lobe epilepsy neurons in humans and a rat model. Exp. Neurol..

[16] Zhang Q., Sano C., Masuda A., Ando R., Tanaka M., Itohara S. (2016). Netrin-G1 regulates fear-like and anxiety-like behaviors in dissociable neural circuits. Sci. Rep..

[17] Zhang W., Rajan I., Savelieva K.V., Wang C.Y., Vogel P., Kelly M., Xu N., Hasson B., Jarman W., Lanthorn T.H. (2008). Netrin-G2 and netrin-G2 ligand are both required for normal auditory responsiveness. Genes Brain Behav..

[18] Aoki-Suzuki M., Yamada K., Meerabux J., Iwayama-Shigeno Y., Ohba H., Iwamoto K., Takao H., Toyota T., Suto Y., Nakatani N. (2005). A family-based association study and gene expression analyses of netrin-G1 and -G2 genes in schizophrenia. Biol. Psychiatry.

[19] Eastwood S.L., Harrison P.J. (2008). Decreased mRNA expression of netrin-G1 and netrin-G2 in the temporal lobe in schizophrenia and bipolar disorder. Neuropsychopharmacology.

[20] Eastwood S.L., Harrison P.J. (2010). Markers of glutamate synaptic transmission and plasticity are increased in the anterior cingulate cortex in bipolar disorder. Biol. Psychiatry.

[21] Borg I., Freude K., Kübart S., Hoffmann K., Menzel C., Laccone F., Firth H., Ferguson-Smith M.A., Tommerup N., Ropers H.H. (2005). Disruption of Netrin G1 by a balanced chromosome translocation in a girl with Rett syndrome. Eur. J. Hum. Genet..

[22] O’Roak B.J., Vives L., Girirajan S., Karakoc E., Krumm N., Coe B.P., Levy R., Ko A., Lee C., Smith J.D. (2012). Sporadic autism exomes reveal a highly interconnected protein network of de novo mutations. Nature.

[23] Wei G., Deng X., Agarwal S., Iwase S., Disteche C., Xu J. (2016). Patient Mutations of the Intellectual Disability Gene KDM5C Downregulate Netrin G2 and Suppress Neurite Growth in Neuro2a Cells. J. Mol. Neurosci..

[24] Zhang Q., Goto H., Akiyoshi-Nishimura S., Prosselkov P., Sano C., Matsukawa H., Yaguchi K., Nakashiba T., Itohara S. (2016). Diversification of behavior and postsynaptic properties by netrin-G presynaptic adhesion family proteins. Mol. Brain.

[25] Nishimura-Akiyoshi S., Niimi K., Nakashiba T., Itohara S. (2007). Axonal netrin-Gs transneuronally determine lamina-specific subdendritic segments. Proc. Natl. Acad. Sci. USA.

[26] Matsukawa H., Akiyoshi-Nishimura S., Zhang Q., Luján R., Yamaguchi K., Goto H., Yaguchi K., Hashikawa T., Sano C., Shigemoto R. (2014). Netrin-G/NGL complexes encode functional synaptic diversification. J. Neurosci..

[27] Abu-Libdeh B., Ashhab M., Shahrour M., Daana M., Dudin A., Elpeleg O., Edvardson S., Harel T. (2019). Homozygous frameshift variant in NTNG2, encoding a synaptic cell adhesion molecule, in individuals with developmental delay, hypotonia, and autistic features. Neurogenetics.

[28] Monies D., Abouelhoda M., Assoum M., Moghrabi N., Rafiullah R., Almontashiri N., Alowain M., Alzaidan H., Alsayed M., Subhani S. (2019). Lessons Learned from Large-Scale, First-Tier Clinical Exome Sequencing in a Highly Consanguineous Population. Am. J. Hum. Genet..

[29] Maussion G., Cruceanu C., Rosenfeld J.A., Bell S.C., Jollant F., Szatkiewicz J., Collins R.L., Hanscom C., Kolobova I., de Champfleur N.M. (2017). Implication of LRRC4C and DPP6 in neurodevelopmental disorders. Am. J. Med. Genet. A..

[30] Sangu N., Shimojima K., Takahashi Y., Ohashi T., Tohyama J., Yamamoto T. (2017). A 7q31.33q32.1 microdeletion including *LRRC4* and *GRM8* is associated with severe intellectual disability and characteristics of autism. Hum. Genome Var..

[31] Um S.M., Ha S., Lee H., Kim J., Kim K., Shin W., Cho Y.S., Roh J.D., Kang J., Yoo T. (2018). NGL-2 Deletion Leads to Autistic-like Behaviors Responsive to NMDAR Modulation. Cell Rep..

[32] Choi Y., Park H., Jung H., Kweon H., Kim S., Lee S.Y., Han H., Cho Y., Kim S., Sim W.S. (2019). NGL-1/LRRC4C Deletion Moderately Suppresses Hippocampal Excitatory Synapse Development and Function in an Input-Independent Manner. Front. Mol. Neurosci..

[33] DeNardo L.A., de Wit J., Otto-Hitt S., Ghosh A. (2012). NGL-2 regulates input-specific synapse development in CA1 pyramidal neurons. Neuron.

[34] Pirone A., Cozzi B., Edelstein L., Peruffo A., Lenzi C., Quilici F., Antonini R., Castagna M. (2012). Topography of Gng2- and NetrinG2-expression suggests an insular origin of the human claustrum. PLoS ONE.

