# Supplementary material for: Homozygous Missense Variants in NTNG2, Encoding a Presynaptic Netrin-G2 Adhesion Protein, Lead to a Distinct Neurodevelopmental Disorder
Source: Am J Hum Genet. 2019 Oct 24;105(5):1048–56. doi: 10.1016/j.ajhg.2019.09.025 (PMC6849109; doi:10.1016/j.ajhg.2019.09.025)
Supplement: Document S3. Article plus Supplemental Data [file mmc7.pdf]

REPORT

Homozygous Missense Variants in *NTNG2*, Encoding a Presynaptic Netrin-G2 Adhesion Protein, Lead to a Distinct Neurodevelopmental Disorder

Caroline M. Dias,[1,2,32] Jaya Punetha,[3,31,32] Céline Zheng,[4,32] Neda Mazaheri,[5,6] Abolfazl Rad,[7,17] Stephanie Efthymiou,[8] Andrea Petersen,[9] Mohammadreza Dehghani,[10] Davut Pehlivan,[3,11] Jennifer N. Partlow,[1,12] Jennifer E. Posey,[3] Vincenzo Salpietro,[8] Alper Gezdirici,[13] Reza Azizi Malamiri,[14] Nihal M. Al Menabawy,[15] Laila A. Selim,[15] Mohammad Yahya Vahidi Mehrjardi,[10] Selina Banu,[16] Daniel L. Polla,[17,18] Edward Yang,[19] Jamileh Rezazadeh Varaghchi,[20] Tadahiro Mitani,[3] Ellen van Beusekom,[17] Maryam Najafi,[21] Alireza Sedaghat,[22] Jennifer Keller-Ramey,[23] Leslie Durham,[9] Zeynep Coban-Akdemir,[3] Ender Karaca,[3,24] Valeria Orlova,[4] Lieke L.M. Schaeken,[17] Amir Sherafat,[25] Shalini N. Jhangiani,[26] Valentina Stanley,[27] Gholamreza Shariati,[6,28] Hamid Galehdari,[5] Joseph G. Gleeson,[27] Christopher A. Walsh,[1,12] James R. Lupski,[3,26,29,30] Elena Seiradake,[4] Henry Houlden,[8] Hans van Bokhoven,[17] and Reza Maroofian[8,*]

NTNG2 encodes netrin-G2, a membrane-anchored protein implicated in the molecular organization of neuronal circuitry and synaptic organization and diversification in vertebrates. In this study, through a combination of exome sequencing and autozygosity mapping, we have identified 16 individuals (from seven unrelated families) with ultra-rare homozygous missense variants in *NTNG2*; these individuals present with shared features of a neurodevelopmental disorder consisting of global developmental delay, severe to profound intellectual disability, muscle weakness and abnormal tone, autistic features, behavioral abnormalities, and variable dysmorphisms. The variants disrupt highly conserved residues across the protein. Functional experiments, including *in silico* analysis of the protein structure, *in vitro* assessment of cell surface expression, and *in vitro* knockdown, revealed potential mechanisms of pathogenicity of the variants, including loss of protein function and decreased neurite outgrowth. Our data indicate that appropriate expression of *NTNG2* plays an important role in neurotypical development.

Based on studies in invertebrates and chicken and mouse, netrins are considered to be the paradigmatic axon guidance molecules, yet the essential role of this family of proteins in humans remains unclear. Members of the classical netrin family are secreted proteins that include UNC6 (uncoordinated-6) in *C. elegans* and netrins NTN1–4 in vertebrates.[1,2] Netrin-G proteins (NTNG1 and NTNG2) are distinct from classical netrins in that they are vertebrate-specific, membrane-bound proteins tethered to the plasma membrane by glycosyl phosphatidylinositol (GPI) anchors.[3] *NTNG1* (MIM: 608818) and *NTNG2* are predominantly expressed in a non-overlapping and complementary pattern in specific neuronal subsets of the developing and mature central nervous system.[4–6] The proteins interact with the extracellular region of their specific netrin-G ligand receptors NGL-1/LRRC4C (MIM: 608817) and NGL-2/LRRC4, respectively.[7] Selectivity in binding between netrin-G molecules and their cognate receptors is

[1]Division of Genetics and Genomics, Boston Children's Hospital, Harvard Medical School, Boston, MA 02115, USA; [2]Division of Developmental Medicine, Boston Children's Hospital, Harvard Medical School, Boston, MA 02115, USA; [3]Department of Molecular and Human Genetics, Baylor College of Medicine, Houston, TX 77030, USA; [4]Department of Biochemistry, University of Oxford, South Parks Road, Oxford, OX1 3QU, UK; [5]Department of Genetics, Faculty of Science, Shahid Chamran University of Ahvaz, Ahvaz, 6135783151, Iran; [6]Narges Medical Genetics and Prenatal Diagnosis Laboratory, Kianpars, Ahvaz, 6155689467, Iran; [7]Cellular and Molecular Research Center, Sabzevar University of Medical Sciences, Sabzevar, 009851, Iran; [8]Department of Neuromuscular Disorders, Queen Square Institute of Neurology, University College London, WC1N 3BG, London, UK; [9]Randall Children's Hospital at Legacy Emanuel, Portland, OR 97227, USA; [10]Medical Genetics Research Centre, Shahid Sadoughi University of Medical Sciences, Yazd, Iran; [11]Section of Pediatric Neurology and Developmental Neuroscience, Department of Pediatrics, Baylor College of Medicine, Houston, TX 77030, USA; [12]Howard Hughes Medical Institute, Boston Children's Hospital, Boston, MA 02115, USA; [13]Department of Medical Genetics, Kanuni Sultan Suleyman Training and Research Hospital, Istanbul, 34303, Turkey; [14]Department of Paediatric Neurology, Golestan Medical, Educational, and Research Center, Ahvaz Jundishapur University of Medical Sciences, Ahvaz 6163764648, Iran; [15]Pediatric Neurology and Metabolic Division, Cairo University Children Hospital, Egypt; [16]Department of Pediatric Neurology, ICH and SSF Hospital Mirpur, Dhaka, 1216, Bangladesh; [17]Department of Human Genetics, Donders Institute for Brain, Cognition and Behaviour, Radboud University Medical Center, 6500 HB, Nijmegen, the Netherlands; [18]CAPES Foundation, Ministry of Education of Brazil, 549 Brasília, Brazil; [19]Department of Radiology, Boston Children's Hospital, Boston, MA 02115, USA; [20]Hasti Genetic Counseling Center of Welfare Organization of Southern Khorasan, Birjand, Iran; [21]Genome Research Division, Human Genetics Department, Radboud University Medical Center, 6500 HB, Nijmegen, the Netherlands; [22]Health Research Institute, Diabetes Research Center, Ahvaz Jundishapur University of Medical Sciences, Ahvaz, Iran; [23]GeneDx, Gaithersburg, MD 20877, USA; [24]Department of Genetics, University of Alabama at Birmingham, Birmingham, AL 35294, USA; [25]Department of Neurology, Faculty of Medicine, Bam University of Medical Sciences, Bam, Iran; [26]Human Genome Sequencing Center, Baylor College of Medicine, Houston, TX 77030, USA; [27]Laboratory for Pediatric Brain Disease, Howard Hughes Medical Institute, Department of Neurosciences, University of California, San Diego, La Jolla, CA 92093, USA; [28]Department of Medical Genetics, Faculty of Medicine, Ahvaz Jundishapur University of Medical Sciences, Ahvaz 6135715794, Iran; [29]Department of Pediatrics, Baylor College of Medicine, Houston, TX 77030, USA; [30]Texas Children's Hospital, Houston, TX 77030, USA
[31]Present address: Department of Genetics and Genomic Sciences, Icahn School of Medicine at Mount Sinai, New York City, NY 10029, USA
[32]These authors contributed equally to this work
*Correspondence: r.maroofian@ucl.ac.uk
https://doi.org/10.1016/j.ajhg.2019.09.025.
© 2019 The Author(s). This is an open access article under the CC BY license (http://creativecommons.org/licenses/by/4.0/).

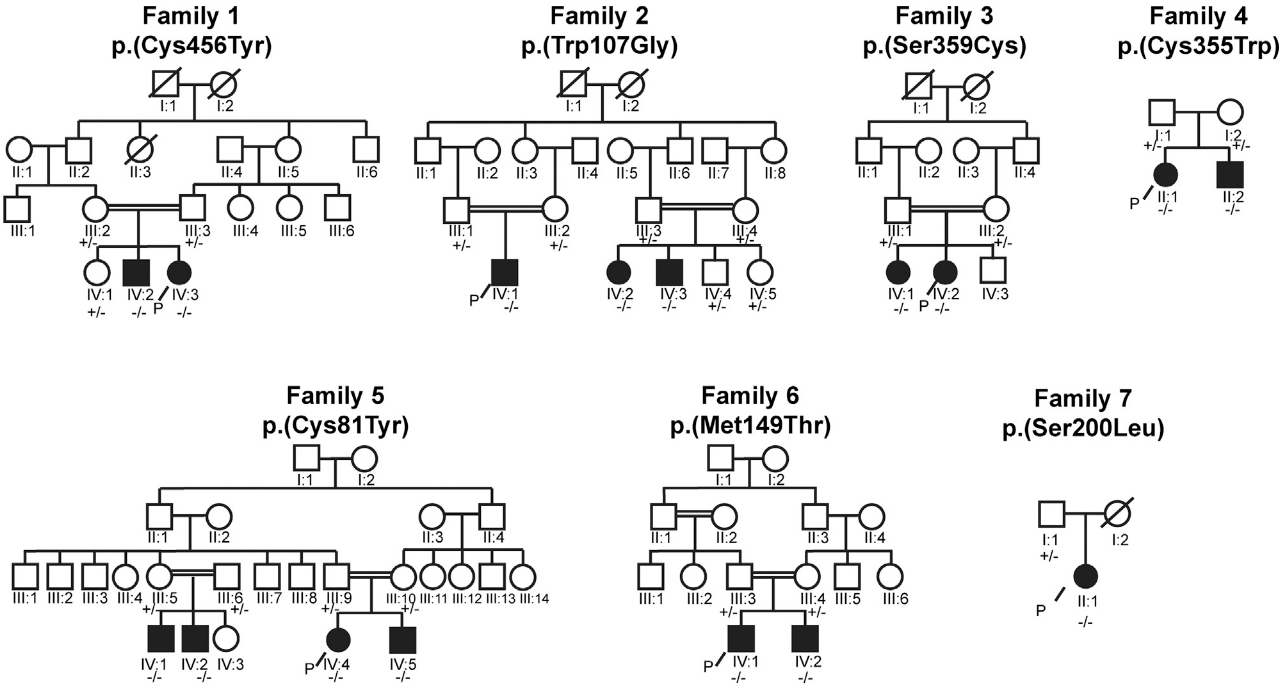

Figure 1. Pedigrees of All Families with Affected Individuals and Variants and Segregation Findings
+ indicates wild-type allele, - indicates variant allele, P indicates proband.

mediated by the interactions of three loops of the laminin domain, and the extracellular leucine rich repeats (LRR) domain of NGLs results in a molecular hand-clasp interaction of high affinity.[8]

NTNG2 encodes netrin-G2, a vertebrate-specific protein that is part of a distinct functional sub-class of the highly conserved netrin family. The netrin family provides axonal guidance cues during central nervous system development.[9] *NTNG2* is located on 9q34.13, and the canonical transcript consists of eight exons including seven coding exons; it encodes a 530-amino-acid protein. *NTNG2* demonstrates evidence of missense constraint in the ExAC database, with a Z score of 4.34, and review of population-based (gnomAD) and ethnically diverse in-house databases reveals an absence of homozygous damaging and/or deleterious variants. Despite this constraint, its potential role in human genetic disease is not clear. Here we show that bi-allelic missense variants in *NTNG2* cause a distinctive neurological and behavioral disorder that highlights the importance of this family of genes in human nervous system development.

We have identified 16 individuals (from seven unrelated families) who have ultra-rare bi-allelic variants in *NTNG2* and who present with shared clinical features of a neurodevelopmental disorder. Consent for clinical data and biological material collection, use, and storage was obtained from all participating families after written informed consent was provided, and studies for each family were approved by their respective institutional review boards (see Supplemental Data for further details). Following genomic DNA extraction from blood, exome sequencing, and homozygosity mapping, we identified 16 individuals in seven unrelated families from different parts of Iran (Families 1, 2, 3), Mexico (Family 4), Turkey (Family 5), Egypt (Family 6), and Bangladesh (Family 7) who have a similar clinical phenotype and have homozygous missense variants in *NTNG2* (Figure S1,Table S1). Researchers and physicians for all families were connected using the GeneMatcher/Matchmaker exchange.[10,11] All families except for Families 4 and 7 had a known history of consanguinity, and all of the variants were segregated in the affected families in accordance with Mendelian expectations for a recessive disease trait (Figure 1). Autozygosity mapping for Families 4 and 7 revealed distant relatedness, and parents of the proband in Family 7 come from the same village (Figure S2, Table S2). There was no evidence of neuropsychiatric disorders in the heterozygous family members presented here.

Clinical features of affected individuals are presented in Table 1. Affected individuals presented with global developmental delay with severe to profound intellectual disability; the majority were non-verbal and non-ambulatory. Most individuals also had features of autism and all were noted to have mood and/or behavioral challenges, many of which were similar to those seen in Rett syndrome, such as hand stereotypy, episodes of laughing and/or screaming, and bruxism, and in Angelman syndrome (Videos S1–S4). Gastrointestinal symptoms, including constipation and bloating, were also common. Growth parameters were below average, and four individuals had documented failure to thrive. Secondary microcephaly was also observed. Dysmorphic features were variable and included low-set ears, hypotelorism, and frontal

Table 1. Clinical Features of Affected Individuals

Family	1	1	2	2	2	3	3	4	4	5	5	5	5	6	6	7
Individual	IV:2	IV:3	IV:1	IV:2	IV:3	IV:1	IV:2	II:1	II:2	IV:1	IV:2	IV:4	IV:5	IV:1	IV:2	II:1
Age (years)	18	10	11	16	9	15	11	11	21	11	1.25	9	5	11	8	3
Sex	M	F	M	M	F	F	F	F	M	M	M	F	M	M	M	F
ID/GDD	+	+	+	+	+	+	+	+	+	+	+	+	+	+	+	+
Motor delay	+	+	+	+	+	+	+	+	+	+	+	+	+	+	+	+
Language delay	+	+	+	+	+	+	+	+	+	+	NA	+	+	+	+	+
Autistic features/stereotypy	+	+	+	+	+	+	+	+	-	+	NA	+	+	+	-	+
Hyperactivity	-	+	+	+	+	-	-	-	+	-	-	-	-	+	+	-
Screaming/laughing spells	+	+	+	+	+	+	+	+	+	+	NA	+	-	+	-	+
Self-injury/hand-biting	+	+	+	+	+	-	-	+	+	-	-	-	-	-	-	-
Bruxism	-	+	+	+	+	-	-	-	-	-	NA	+	+	+	-	-
Hypotonia in infancy	+	+	+	+	+	+	+	+	+	+	+	+	+	-	-	+
Nonambulatory	+	+	+	+	+	+	+	+	+	+	NA	+	+	-	-	-
Brain imaging abnormalities	-	NA	+	NA	NA	-	+	-	NA	NA	NA	+	+	+	+	+
Seizures	-	-	+	-	-	-	-	+	+	-	-	-	-	+	-	+
Microcephaly	-	+	-	+	+	-	-	+	NA	NA	-	+	-	-	-	-
Secondary Microcephaly		+		NA	+			NA				NA				
Dysmorphic features	-	-	+	-	-	+	+	-	-	NA	+	+	+	+	+	-
Ophthalmologic features	-	-	-	-	-	+	+	-	-	-	-	+	+	-	-	-
GI symptoms	+	+	+	+	+	+	+	+	+	+	+	+	+	-	-	+

ID/GDD, intellectual disability/global developmental delay; GI, gastrointestinal; -, absent; +, present; NA, not ascertained/not applicable

bossing (Figure 2A). Neurologically, hypotonia in infancy and muscle weakness and/or atrophy were common findings. Five individuals had early-onset seizures, and four were noted to have ocular findings of esotropia, nystagmus, or strabismus. Brain imaging, conducted in both infancy and childhood, demonstrated findings ranging from normal to mild brain atrophy with white matter abnormalities (Figure 2B). In summary, affected individuals display a neurodevelopmental disorder of severe-to-profound intellectual disability with marked motor involvement and mood and behavioral challenges including autistic features, as well as poor growth and facial dysmorphisms. Detailed phenotypic descriptions are provided in Table 1, Table S1, Figure 2, and the Supplemental Note: Case Reports in Supplemental Data.

The ultra-rare variants we identified from family based genomic studies in the above individuals were notable for several reasons (Table S2). All variants were absent from both local ethnically diverse in-house databases, as well as large population databases. Because most of the *NTNG2* missense variants observed are rare to their specific "clan," they may reflect variants that arose recently and according to the clan genomics hypothesis are therefore expected to have a larger influence on disease.[12] All variants were predicted by a majority of prediction tools (FATHMM, MutationAssessor, MutationTaster, PolyPhen-2, SIFT, PROVEAN, and CADD) to be likely damaging to protein function, and genomic evolutionary rate profiling (GERP) indicated that these sites may be under evolutionary constraint (Table S2). In fact, all variants impact residues conserved in NTNG1, a result that gives further evidence for the argument that they are pathogenic. Annotation of the variant locations on the protein domains of NTNG2 revealed that they are not confined to one domain, but they fall within the laminin and EGF domains and are predicted to disrupt structural motifs within NTNG2 (Figure 3A). No other alternative candidate variants common to the families were identified (Table S3).

The available NGL2/netrin-G2 crystal structure contains a model of the netrin-G2 N terminus up to the first EGF domain. We used MODELER[13] to create a homology for the EGF domains 2–4, which were not included in that crystal structure. Using these models, we found that the variants are located in the laminin, EGF2, or EGF4 domain (Figure 3B–3C, Figure S3). In addition to possible effects on specific protein-to-protein interaction sites, this suggests a more global mechanism of functional disruption. Strikingly, we found that four of the seven, i.e., 57% of the variants, involve the loss or addition of cysteine residues (GenBank: NM_032536.3: c.242G>A [p.Cys81Tyr], c.1065C>G

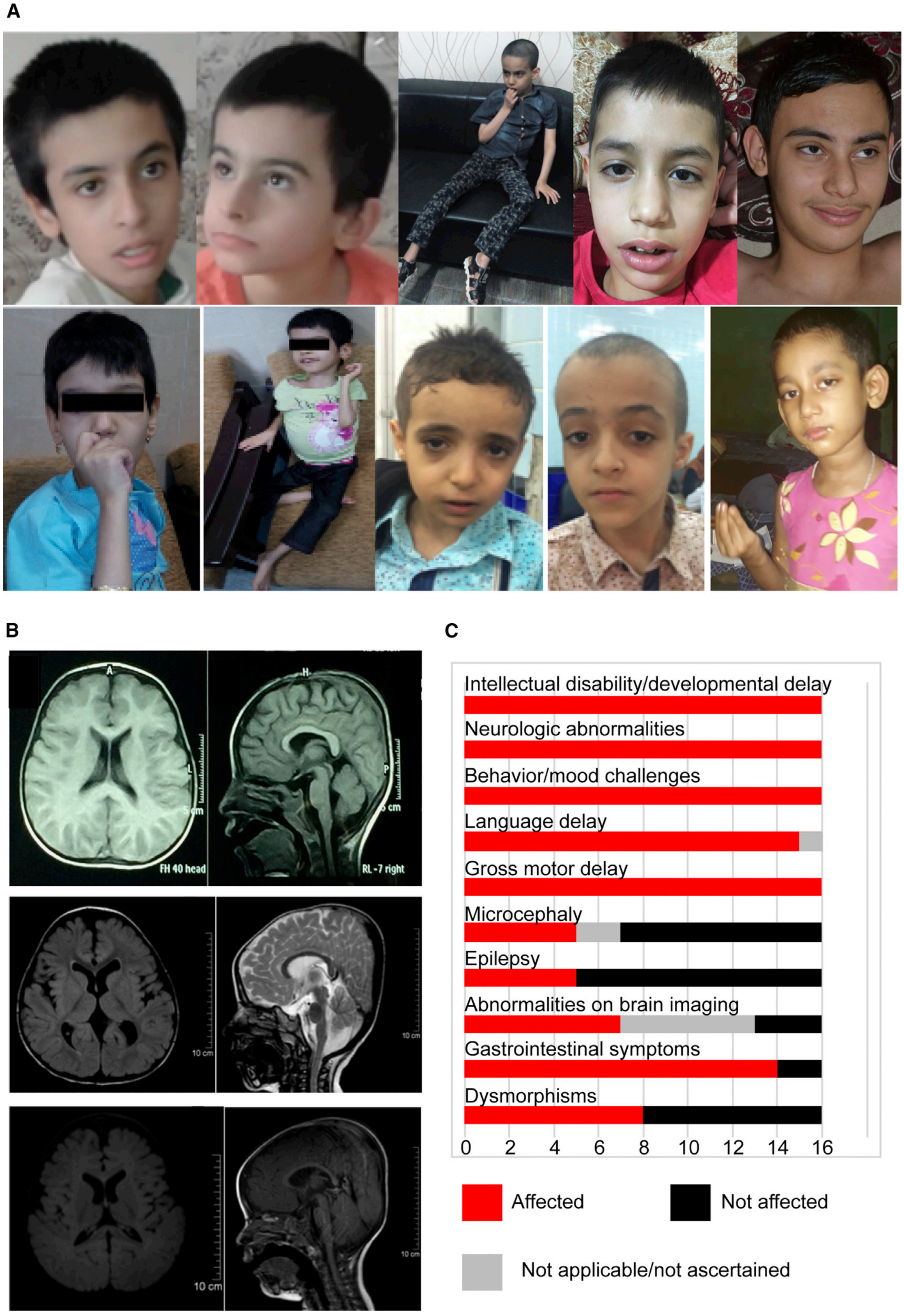

Figure 2. Clinical Features of Affected Individuals

(A) Representative photographs demonstrating clinical features of affected individuals; these features include facial features, muscular atrophy, and hand stereotypy. Top row from left to right: Family 2 IV:2, IV:3, IV:1; Family 1 IV:3, IV:2. Bottom row: Family 3 IV:1, IV:2; Family 6 IV:2, IV:1; Family 7 II:1.

(B) Representative MRIs of affected individuals, demonstrating decreased brain volume. From top to bottom: Family 6 IV:2; Family 5 IV:4; Family 5 IV:5.

(C) Bar graph summarizing proportions of various clinical findings affecting individuals.

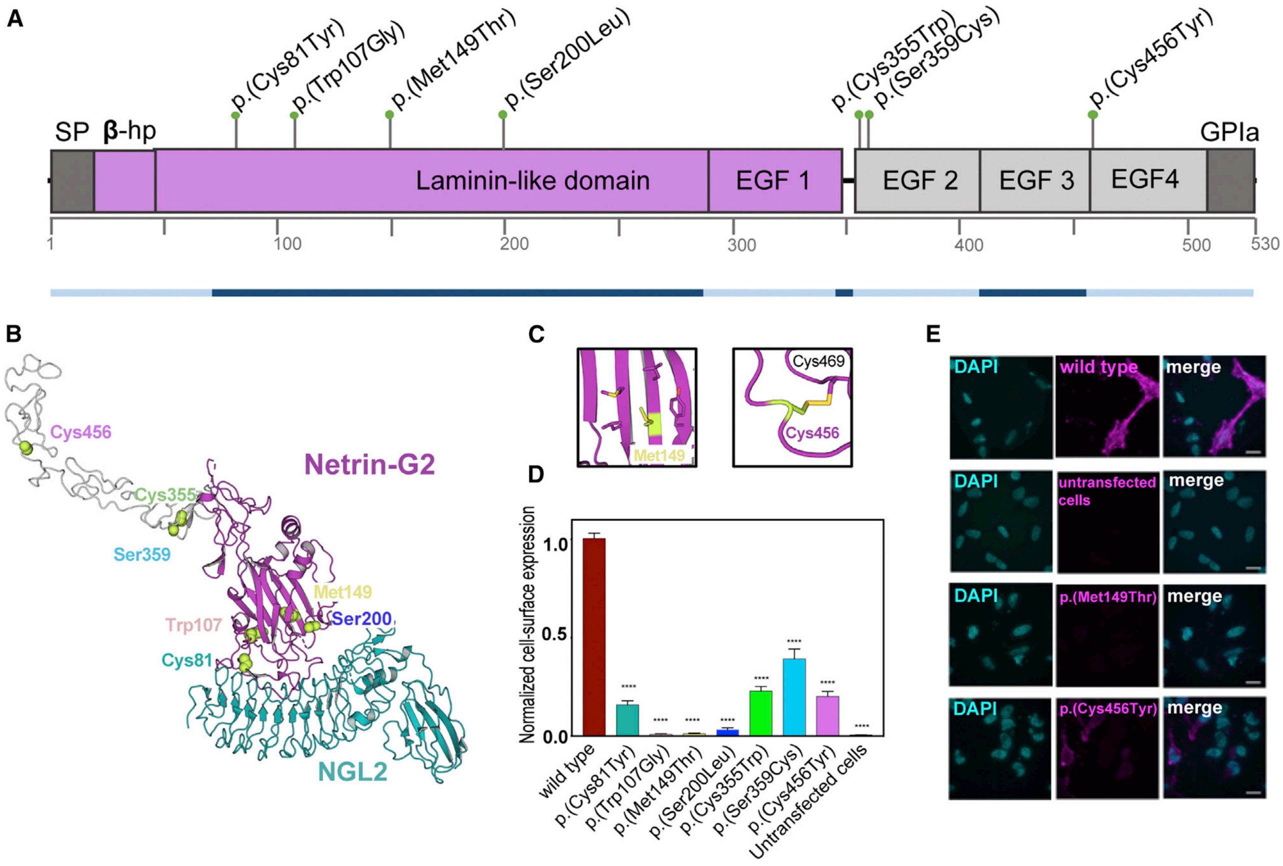

Figure 3. Structural Mapping and Cell Surface Expression

(A) Netrin-G2 domain overview. The positions of altered residues relative to the protein domains are indicated. Domain nomenclature is: SP, signal peptide; β-hp, N-terminal β-hairpin domain; EGF, epidermal-growth factor like; GPIa, GPI anchor. Corresponding exons are represented underneath the domain organization in blue.

(B) Full-length model of netrin-G2 based on the crystal structure of the Laminin-like domain and EGF1[8] (purple) and on homology models of EGF2-4 (gray), in complex with its ligand NGL2 (cyan). The residues that are mutated in the presented variants are indicated as green spheres.

(C) Close-up views of Met149 and Cys456 residues as found in the structural model shown in panel B. For close-up views of the other mutated residues, see Figure S3.

(D) The quantification of the cell surface expression levels of wild type (WT) and mutant netrin-G2 constructs (see panel E) is shown (mean ± SEM). The variants show significantly reduced cell-surface expression compared to the WT (****$p < 0.0001$).

(E) Netrin-G2 constructs were expressed in HeLa cells with an N-terminal flag tag. Flag-tagged protein was detected via cell-surface immunostaining (magenta). DAPI (blue) highlights cell nuclei. Representative images are shown for WT netrin-G2, untransfected cells (negative control), p.Met149Thr and p.Cys456Tyr variants. Representative images of other variants are shown in Figure S3. Scale bar is 15 μm.

[p.Cys355Trp], c.1076C>G [p.Ser359Cys], c.1367G>A [p.Cys456Tyr]). Given that the cysteine content of NTNG2 is only 7.9% in humans, the enrichment for cysteine variants in this cohort suggests a mechanism of pathogenicity. Due to the oxidizing environment in the endoplasmic reticulum (ER) and extracellular space, cysteine residues found in extracellular proteins typically appear in pairs and form disulfide bridges. Such bridges can stabilize a protein by reducing the entropy of the unfolded state and/or they can facilitate the path to the native state if they link parts of a protein that must come into contact early during a folding reaction.[14] Unpaired exposed cysteines are detected by the ER quality control machinery and targeted for refolding or degradation.[14] We hypothesize that the *NTNG2* variants involving cysteine could have a negative effect on protein stability and cell surface expression.

Three out of the seven variants do not involve cysteines: c.599C>T (p.Ser200Leu), c.319T>G (p.Trp107Gly), and c.446T>C, (p.Met149Thr). For both p.Trp107Gly and p.Met149Thr, a large hydrophobic residue (Trp or Met) is changed to either one lacking a side chain (Gly) or one bearing a small polar side chain (Thr). Both of these residues form part of the hydrophobic core that stabilizes the folding of the netrin-G2 laminin domain. Disruption likely causes protein misfolding and lack of expression at the cell surface. Thus the consequence of both types of variants, cysteine-dependent or hydrophobic core disruptive, is potentially a similar reduction in protein stability and expression at the cell surface. The p.Ser200Leu variant does not fit into either of the above categories, with Ser200 located at the periphery of the laminin domain. It is also located ~0.9 nm away from the surface of NGL2

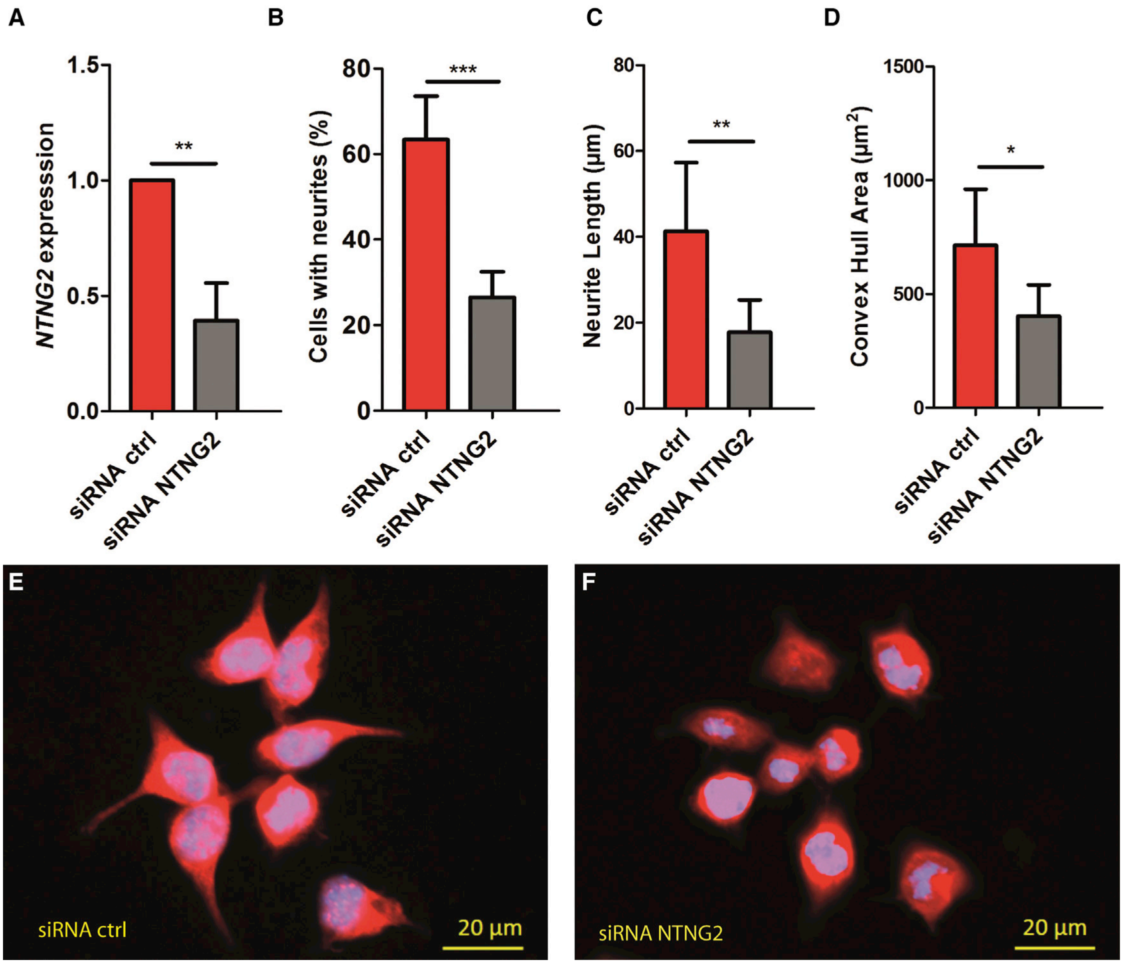

Figure 4. *Ntng2* Knockdown in N2a Cells

(A) Knockdown of endogenous *Ntng2* by *Ntng2*-specific siRNAs as normalized to control siRNA. Results of quantitative RT-PCR 30 h post-transfection.

(B–D) Effects of *Ntng2* knockdown on neurite outgrowth. Data presented as mean and SD; $*p < 0.05$, $**p < 0.01$, $***p < 0.001$. Quantification was conducted by counting the absolute number of cells with neurites (B), measuring the neurite length by NeuroLucida tracing (C), and quantification of the Convex hull area (D). All analyses show a significant reduction of neurite number and length as a consequence of *Ntng2* knockdown.

(E–F) Example of the N2a appearance at 30 h post-transfection with control (E) and *Ntng2*-specific siRNA (F). Visualization was done using MAP2 counterstaining (red).

as found in the crystal structure (Figure S3). With typical hydrogen bonds about 0.25 nm in length, Ser200 is not interacting directly with NGL2, although allosteric effects could still influence the netrin-G2 binding loops.

We tested all variants for function by overexpressing wild-type (WT) and variant netrin-G2 constructs in HeLa cells and assessing their presence at the cell surface through the use of indirect immunofluorescence and immunoblotting validation (Figure S4). All variants displayed substantially decreased cell surface expression as compared to WT (Figures 3D–3E). Notably, some of these variants had more cell surface expression compared to others, suggesting that some netrin-G2 may still be localized in these individuals. The variants may nevertheless show deficient ligand-receptor binding or signaling since we did not observe a clear association with cell surface expression levels and clinical phenotype severity.

Given the decreased cell surface expression pattern observed in all seven variants, we sought to determine the more global effects of *NTNG2* loss of function. Using siRNA to target endogenous *Ntng2* expression in mouse N2A cells, we first confirmed that transfection with our *Ntng2*-specific siRNA led to decreased expression with quantitative polymerase chain reaction (Figure 4A). We next assessed neurite outgrowth and found a significant reduction for all parameters assessed, which included neurite number, neurite length, and convex hull area—a measurement used for measuring dendritic field (Figures 4B–4F). These findings demonstrate a potential mechanism by which the *NTNG2* variants may contribute to pathological neurodevelopment.

Netrin signaling has been implicated in neurologic and psychiatric disorders. For example, conditional *Ntng1* knockout in distinct neuronal subtypes is associated with

alterations in fear and anxiety-like behaviors in rodent models, and abnormal expression of *NTNG2* has been found in the human brain in refractory epilepsy.[15,16] Studies of *Ntng2* and *Ngl2* knockout mice have shown that both types of mutant mice have an identical phenotype of lack of behavioral startle in response to acoustic stimulus, with no structural abnormalities noted in the inner ear.[17] Single-nucleotide polymorphisms (SNPs) and differential expression patterns of *NTNG1* and *NTNG2* have been associated with schizophrenia and bipolar disorder in humans.[18–20] A *de novo* genomic rearrangement involving *NTNG1* was proposed to potentially cause features of Rett syndrome in an isolated individual.[21] Additionally, *de novo* missense variants in *NTNG1* were reported in two individuals with autism spectrum disorder.[22] An *in vitro* study of variants in the histone demethylase *KDM5C* (Lysine demethylase 5C [MIM: 314690]), which is known to cause intellectual disability, showed that *NTNG2* seemed to be important in mediating effects on neurite growth and length; these results are consistent with our findings here.[23] In fact, several clinical features, in addition to intellectual disability, are shared between these two disorders, including variable neurologic, behavioral, and dysmorphic features. Extensive behavioral battery on *Ntng2* knockout mice demonstrated marked deficits in learning, memory, and visual and motor functioning.[24] Although *NTNG2* does not appear to be necessary for axon guidance, it has been shown to be important in the laminar distribution of its receptors and synaptic plasticity.[25,26] A homozygous founder frameshift variant in *NTNG2* has recently been identified in eight individuals from four families with a similar clinical phenotype, and this further strengthens the evidence supporting the pathogenicity of the variants presented here.[27]

Other genes involved in netrin signaling have also been implicated in neurodevelopmental disorders in isolated case reports, some of which have involved examples of *de novo* variation.[28] Specifically, variations in *LRRC4C* and *LRRC4* have both been associated with intellectual disability and autism.[29,30] Furthermore, functional work in mice has shown that *LRRC4* expression regulates N-methyl-D-aspartate receptor (NMDAR)-dependent synaptic plasticity and prevents autistic-like behaviors.[31] *LRRC4C* and *LRRC4* have both been shown to be important in hippocampal synapse formation and function.[32,33] The marked findings of severe intellectual disability and autistic features in our cohort are particularly intriguing given the unique role of *NTNG2* in vertebrates. As we previously mentioned, netrin-g family members express in distinct, non-overlapping, and complementary neuronal circuits, suggesting a role in establishing appropriate neuronal patterning. This neuronal compartmentalization parallels distinct behavioral compartmentalization, as in mouse knockout models, *Ntng2* knockouts demonstrated sensorimotor, spatial memory, working memory, procedural learning, and attentional deficits, while *Ntng1* knockouts demonstrated distinct learning and fear conditioning deficits.[24] Our findings here, in conjunction with the known role of NTNG2 in the control of synaptic plasticity and postsynaptic membrane organization, illustrate the clinical relevance of these neuronal functions to higher cognitive processes. In fact, given the profound finding of intellectual disability in the individuals presented here, it is intriguing that *NTNG2* expression is enriched in the human claustrum, an enigmatic brain region posited to play a role in the integration of conscious perception.[34]

Our work provides the groundwork for establishing a genotype-to-phenotype relationship with *NTNG2* variants, and establishes an initial description of the clinical spectrum. *NTNG2* should be considered in the clinical evaluation of children with severe intellectual disability and neuropsychiatric symptoms. In addition to identification by exome sequencing, it will be important to add *NTNG2* to clinical gene-panel tests for intellectual disability given the marked yet variable clinical phenotype. In summary, our results implicate rare bi-allelic missense *NTNG2* variants in the pathobiology of a neurodevelopmental disorder consisting of severe intellectual disability, autistic features, and motor impairment. Our findings provide strong clinical and functional evidence for the importance of the appropriate expression of *NTNG2* in neurodevelopment.

Accession Numbers

The accession numbers for the variants reported in this paper are ClinVar SCV000994967.1, SCV000994968.1, SCV000994969.1, SCV000994970.1, SCV000994971.1, SCV000994972.1, and SCV000994973.1.

LOVD variant identification numbers are 597120–597126.

Supplemental Data

Supplemental Data can be found online at https://doi.org/10.1016/j.ajhg.2019.09.025.

Acknowledgments

This work was supported by the United States National Institutes of Health: U54HG003067 (E.L.), UM1HG008900 (D.M., H.R.), U54HG006504 (R.L., M.G.), R35 NS105078 (J.R.L.), UM1HG006542 (J.R.L.), K08HG008986 (J.E.P.), R01NS048453 (J.G.G.), R01NS052455 (J.G.G.), T32 NS043124-17 (D.P.), R01NS035129 (C.A.W.), T32 MH112510 (C.M.D.). Additional support includes the Muscular Dystrophy Association (#512848 [J.R.L]., H.H.); the Clinical Research Training Scholarship in Neuromuscular Disease partnered by American Brain Foundation and Muscle Study Group (D.P.); Howard Hughes Medical Institute (C.A.W., J.G.G.); Wellcome Trust (202827/Z/16/Z, [E.S.], Wellcome Trust DPhil. Cellular Structural Biology [C.Z.]; SYNaPS Study Group and Synaptopathies Strategic Award 165908, WT093205 MA, WT104033AIA [H.H].); National Institute for Health Research University College London Hospitals Biomedical Research Centre (H.H.); Rosetree Trust (H.H.); Ataxia UK (H.H.); MSA Trust (H.H.); Brain Research UK (H.H.); Muscular Dystrophy UK (H.H.); Higher Education Commission of Pakistan (H.H.); The MRC (MR/S01165X/1, MR/S005021/1, G0601943 [H.H.]); European Union

Seventh Framework Program (Gencodys: 241995 [H.vB.]); CAPES Fellowship, Brazil (99999.013311/2013-01 [D.L.P.]); Uehara Memorial Foundation (T.M.); the Broad Institute of MIT; Yale Center for Mendelian Disorders; Harvard Center for Mendelian Disorders; and Queen Square Genomics group at University College London. Genome sequencing was performed through an in-kind donation of sequencing from Human Longevity Inc., and from the Rady Children's Institute of Genomic Medicine.

Declaration of Interests

Baylor College of Medicine (BCM) and Miraca Holdings have formed a joint venture with shared ownership and governance of Baylor Genetics (BG), which performs clinical microarray analysis and clinical exome sequencing. J.R.L. serves on the Scientific Advisory Board of BG. J.R.L. has stock ownership in 23andMe, is a paid consultant for Regeneron Pharmaceuticals, has stock options in Lasergen, and is a co-inventor on multiple United States and European patents related to molecular diagnostics for inherited neuropathies, eye diseases, and bacterial genomic fingerprinting. The other authors declare no competing interests.

Received: June 17, 2019
Accepted: September 25, 2019
Published: October 24, 2019

Web Resources

CADD, https://cadd.gs.washington.edu/
ExAC, http://exac.broadinstitute.org/
FATHMM web server, http://fathmm.biocompute.org.uk/
GeneMatcher, https://genematcher.org/
GERP, http://mendel.stanford.edu/sidowlab/downloads/gerp/index.html
gnomAD, https://gnomad.broadinstitute.org/
MutationAssessor web server, http://mutationassessor.org/r3/
MutationTaster, http://www.mutationtaster.org/
Online Mendelian Inheritance in Man, https://www.omim.org
PolyPhen-2, http://genetics.bwh.harvard.edu/pph2/
PROVEAN, http://provean.jcvi.org/index.php
SIFT, https://sift.bii.a-star.edu.sg/

References

1. Serafini, T., Kennedy, T.E., Galko, M.J., Mirzayan, C., Jessell, T.M., and Tessier-Lavigne, M. (1994). The netrins define a family of axon outgrowth-promoting proteins homologous to C. elegans UNC-6. Cell *78*, 409–424.
2. Dickson, B.J. (2002). Molecular mechanisms of axon guidance. Science *298*, 1959–1964.
3. Cirulli, V., and Yebra, M. (2007). Netrins: beyond the brain. Nat. Rev. Mol. Cell Biol. *8*, 296–306.
4. Nakashiba, T., Nishimura, S., Ikeda, T., and Itohara, S. (2002). Complementary expression and neurite outgrowth activity of netrin-G subfamily members. Mech. Dev. *111*, 47–60.
5. Meerabux, J.M., Ohba, H., Fukasawa, M., Suto, Y., Aoki-Suzuki, M., Nakashiba, T., Nishimura, S., Itohara, S., and Yoshikawa, T. (2005). Human netrin-G1 isoforms show evidence of differential expression. Genomics *86*, 112–116.
6. Lin, J.C., Ho, W.H., Gurney, A., and Rosenthal, A. (2003). The netrin-G1 ligand NGL-1 promotes the outgrowth of thalamocortical axons. Nat. Neurosci. *6*, 1270–1276.
7. Woo, J., Kwon, S.K., and Kim, E. (2009). The NGL family of leucine-rich repeat-containing synaptic adhesion molecules. Mol. Cell. Neurosci. *42*, 1–10.
8. Seiradake, E., Coles, C.H., Perestenko, P.V., Harlos, K., McIlhinney, R.A., Aricescu, A.R., and Jones, E.Y. (2011). Structural basis for cell surface patterning through NetrinG-NGL interactions. EMBO J. *30*, 4479–4488.
9. Nakashiba, T., Ikeda, T., Nishimura, S., Tashiro, K., Honjo, T., Culotti, J.G., and Itohara, S. (2000). Netrin-G1: a novel glycosyl phosphatidylinositol-linked mammalian netrin that is functionally divergent from classical netrins. J. Neurosci. *20*, 6540–6550.
10. Philippakis, A.A., Azzariti, D.R., Beltran, S., Brookes, A.J., Brownstein, C.A., Brudno, M., Brunner, H.G., Buske, O.J., Carey, K., Doll, C., et al. (2015). The Matchmaker Exchange: a platform for rare disease gene discovery. Hum. Mutat. *36*, 915–921.
11. Sobreira, N., Schiettecatte, F., Valle, D., and Hamosh, A. (2015). GeneMatcher: a matching tool for connecting investigators with an interest in the same gene. Hum. Mutat. *36*, 928–930.
12. Lupski, J.R., Belmont, J.W., Boerwinkle, E., and Gibbs, R.A. (2011). Clan genomics and the complex architecture of human disease. Cell *147*, 32–43.
13. Webb, B., and Sali, A. (2016). Comparative Protein Structure Modeling Using MODELLER. Curr. Protoc. Bioinformatics. *15*, 5.6.1–5.6.30.
14. Feige, M.J., and Hendershot, L.M. (2011). Disulfide bonds in ER protein folding and homeostasis. Curr. Opin. Cell Biol. *23*, 167–175.
15. Pan, Y., Liu, G., Fang, M., Shen, L., Wang, L., Han, Y., Shen, D., and Wang, X. (2010). Abnormal expression of netrin-G2 in temporal lobe epilepsy neurons in humans and a rat model. Exp. Neurol. *224*, 340–346.
16. Zhang, Q., Sano, C., Masuda, A., Ando, R., Tanaka, M., and Itohara, S. (2016). Netrin-G1 regulates fear-like and anxiety-like behaviors in dissociable neural circuits. Sci. Rep. *6*, 28750.
17. Zhang, W., Rajan, I., Savelieva, K.V., Wang, C.Y., Vogel, P., Kelly, M., Xu, N., Hasson, B., Jarman, W., and Lanthorn, T.H. (2008). Netrin-G2 and netrin-G2 ligand are both required for normal auditory responsiveness. Genes Brain Behav. *7*, 385–392.
18. Aoki-Suzuki, M., Yamada, K., Meerabux, J., Iwayama-Shigeno, Y., Ohba, H., Iwamoto, K., Takao, H., Toyota, T., Suto, Y., Nakatani, N., et al. (2005). A family-based association study and gene expression analyses of netrin-G1 and -G2 genes in schizophrenia. Biol. Psychiatry *57*, 382–393.
19. Eastwood, S.L., and Harrison, P.J. (2008). Decreased mRNA expression of netrin-G1 and netrin-G2 in the temporal lobe in schizophrenia and bipolar disorder. Neuropsychopharmacology *33*, 933–945.
20. Eastwood, S.L., and Harrison, P.J. (2010). Markers of glutamate synaptic transmission and plasticity are increased in the anterior cingulate cortex in bipolar disorder. Biol. Psychiatry *67*, 1010–1016.
21. Borg, I., Freude, K., Kübart, S., Hoffmann, K., Menzel, C., Laccone, F., Firth, H., Ferguson-Smith, M.A., Tommerup, N., Ropers, H.H., et al. (2005). Disruption of Netrin G1 by a balanced chromosome translocation in a girl with Rett syndrome. Eur. J. Hum. Genet. *13*, 921–927.

22. O'Roak, B.J., Vives, L., Girirajan, S., Karakoc, E., Krumm, N., Coe, B.P., Levy, R., Ko, A., Lee, C., Smith, J.D., et al. (2012). Sporadic autism exomes reveal a highly interconnected protein network of de novo mutations. Nature *485*, 246–250.
23. Wei, G., Deng, X., Agarwal, S., Iwase, S., Disteche, C., and Xu, J. (2016). Patient Mutations of the Intellectual Disability Gene KDM5C Downregulate Netrin G2 and Suppress Neurite Growth in Neuro2a Cells. J. Mol. Neurosci. *60*, 33–45.
24. Zhang, Q., Goto, H., Akiyoshi-Nishimura, S., Prosselkov, P., Sano, C., Matsukawa, H., Yaguchi, K., Nakashiba, T., and Itohara, S. (2016). Diversification of behavior and postsynaptic properties by netrin-G presynaptic adhesion family proteins. Mol. Brain *9*, 6.
25. Nishimura-Akiyoshi, S., Niimi, K., Nakashiba, T., and Itohara, S. (2007). Axonal netrin-Gs transneuronally determine lamina-specific subdendritic segments. Proc. Natl. Acad. Sci. USA *104*, 14801–14806.
26. Matsukawa, H., Akiyoshi-Nishimura, S., Zhang, Q., Luján, R., Yamaguchi, K., Goto, H., Yaguchi, K., Hashikawa, T., Sano, C., Shigemoto, R., et al. (2014). Netrin-G/NGL complexes encode functional synaptic diversification. J. Neurosci. *34*, 15779–15792.
27. Abu-Libdeh, B., Ashhab, M., Shahrour, M., Daana, M., Dudin, A., Elpeleg, O., Edvardson, S., and Harel, T. (2019). Homozygous frameshift variant in NTNG2, encoding a synaptic cell adhesion molecule, in individuals with developmental delay, hypotonia, and autistic features. Neurogenetics *20*, 209–213. Epub ahead of print. https://doi.org/10.1007/s10048-019-00583-4.
28. Monies, D., Abouelhoda, M., Assoum, M., Moghrabi, N., Rafiullah, R., Almontashiri, N., Alowain, M., Alzaidan, H., Alsayed, M., Subhani, S., et al. (2019). Lessons Learned from Large-Scale, First-Tier Clinical Exome Sequencing in a Highly Consanguineous Population. Am. J. Hum. Genet. *104*, 1182–1201.
29. Maussion, G., Cruceanu, C., Rosenfeld, J.A., Bell, S.C., Jollant, F., Szatkiewicz, J., Collins, R.L., Hanscom, C., Kolobova, I., de Champfleur, N.M., et al. (2017). Implication of LRRC4C and DPP6 in neurodevelopmental disorders. Am. J. Med. Genet. A. *173*, 395–406.
30. Sangu, N., Shimojima, K., Takahashi, Y., Ohashi, T., Tohyama, J., and Yamamoto, T. (2017). A 7q31.33q32.1 microdeletion including *LRRC4* and *GRM8* is associated with severe intellectual disability and characteristics of autism. Hum. Genome Var. *4*, 17001.
31. Um, S.M., Ha, S., Lee, H., Kim, J., Kim, K., Shin, W., Cho, Y.S., Roh, J.D., Kang, J., Yoo, T., et al. (2018). NGL-2 Deletion Leads to Autistic-like Behaviors Responsive to NMDAR Modulation. Cell Rep. *23*, 3839–3851.
32. Choi, Y., Park, H., Jung, H., Kweon, H., Kim, S., Lee, S.Y., Han, H., Cho, Y., Kim, S., Sim, W.S., et al. (2019). NGL-1/LRRC4C Deletion Moderately Suppresses Hippocampal Excitatory Synapse Development and Function in an Input-Independent Manner. Front. Mol. Neurosci. *12*, 119.
33. DeNardo, L.A., de Wit, J., Otto-Hitt, S., and Ghosh, A. (2012). NGL-2 regulates input-specific synapse development in CA1 pyramidal neurons. Neuron *76*, 762–775.
34. Pirone, A., Cozzi, B., Edelstein, L., Peruffo, A., Lenzi, C., Quilici, F., Antonini, R., and Castagna, M. (2012). Topography of Gng2- and NetrinG2-expression suggests an insular origin of the human claustrum. PLoS ONE *7*, e44745.

The American Journal of Human Genetics, Volume *105*

Supplemental Data

Homozygous Missense Variants in *NTNG2,* Encoding a Presynaptic Netrin-G2 Adhesion Protein, Lead to a Distinct Neurodevelopmental Disorder

Caroline M. Dias, Jaya Punetha, Céline Zheng, Neda Mazaheri, Abolfazl Rad, Stephanie Efthymiou, Andrea Petersen, Mohammadreza Dehghani, Davut Pehlivan, Jennifer N. Partlow, Jennifer E. Posey, Vincenzo Salpietro, Alper Gezdirici, Reza Azizi Malamiri, Nihal M. Al Menabawy, Laila A. Selim, Mohammad Yahya Vahidi Mehrjardi, Selina Banu, Daniel L. Polla, Edward Yang, Jamileh Rezazadeh Varaghchi, Tadahiro Mitani, Ellen van Beusekom, Maryam Najafi, Alireza Sedaghat, Jennifer Keller-Ramey, Leslie Durham, Zeynep Coban-Akdemir, Ender Karaca, Valeria Orlova, Lieke L.M. Schaeken, Amir Sherafat, Shalini N. Jhangiani, Valentina Stanley, Gholamreza Shariati, Hamid Galehdari, Joseph G. Gleeson, Christopher A. Walsh, James R. Lupski, Elena Seiradake, Henry Houlden, Hans van Bokhoven, and Reza Maroofian

Supplemental Note: Case Reports

Family 1

Family 1 is a Persian family with two affected children born to consanguineous parents who are first cousins. The female proband (IV:3), 10 years old at the time of evaluation, was born at full term and weighed 2.8 kg (-0.6 standard deviations (SD)) with a head circumference of 35 cm (+0.2 SD). She was delayed in her early developmental milestones and first walked at 2.5 years; she remains nonverbal with severe intellectual disability (IQ 30). Growth parameters at evaluation include height +0.7 SD, weight at -0.8 SD, and OFC at -2.1 SD. Neurologically, she demonstrated hypotonia in infancy and at evaluation was noted to have tremor, ataxia and muscle weakness. Behaviorally, she demonstrated autism, hand and facial stereotypies, spells of laughing and screaming, anxiety, body rocking, agitation, sleep problems, occasional aggression, hyperactivity, bruxism and hand-biting. Clonidine and risperidone were used for pharmacologic treatment of her behavioral challenges. She had an abnormal EEG but seizure history is unknown. Karyotyping, CytoSNP-array and metabolic testing were normal.

The affected brother (IV:2), age 18 years at evaluation, was born at full term and weighed 3.2 kg (+0.1 SD). Head circumference was reported to be within normal limits. He sat unsupported at about 9-10 months and was delayed in the rest of his early developmental milestones and remained nonambulatory and nonverbal with severe intellectual disability (IQ 25). Growth parameters at evaluation include height at -0.4 SD, weight at -2 SD and head circumference at -1.4 SD. Neurologically, he had hypotonia in infancy and at evaluation was noted to have tremor and muscle weakness. Behaviorally, he demonstrated autism, hand and facial stereotypies, spells of screaming and laughing, anxiety, body rocking, agitation, sleep problems, occasional aggression, and hand-biting. Seizure history is unknown and EEG was reportedly normal. No specific findings on brain MRI at age 12 years were noted. Karyotyping, CytoSNP-array and metabolic testing were normal.

Family 2

Family 2 is an extended consanguineous Bakhtiari (one of the main tribes in southwestern Iran) family with three affected children in two branches. The parents of each branch are first cousins. The proband (IV:1), an 11-year-old boy, was born at full term with a head circumference of 35 cm (+0.6 SD) and weight of 3.65 kg (+0.9 SD). His development was delayed and he remained nonverbal and non-ambulatory with profound intellectual disability. His growth parameters at the time of evaluation were: height at -2.2 SD, weight at -1.8 SD and OFC at -0.9 SD. During neurologic examination, he was hypotonic and had dysphagia, muscle weakness and atrophy, and the DTRs were absent. Seizures began at age 5 and were controlled with valproic acid. Behaviorally, he had autism, and demonstrated hand and facial stereotypies, laughing spells, anxiety and mood changes, body rocking, agitation, sleep problems, hyperactivity, bruxism, and hand biting. Risperidone and melatonin were used with good effect. His EEG was abnormal. His EMG was normal at age 1 year. His CT scan at 9 months showed mild cortical atrophy while two MRIs at 1 year and 6 years revealed diffuse myelination abnormalities. Karyotype analysis at age 9 months revealed a normal male (46, XY) karyotype.

The other two affected children (IV:2, IV:3) are siblings. IV:2, a 16-year-old boy, was born at full term weighing 2.2 kg (-2.2 SD) with a head circumference of 30 cm (-2.7) and was delayed in all aspects of development. He sat unsupported at approximately 18 months of age but remained nonambulatory and nonverbal with profound intellectual disability. Growth parameters at the time of evaluation were: height at -4.4 SD, weight at -6.0 SD and OFC at -2.1 SD. During neurologic examination, he was hypotonic and had muscle weakness and atrophy. He had no history of seizures. Behaviorally, he had autism and demonstrated hand and facial

stereotypy, laughing spells, anxiety and mood changes, agitation, sleep problems, hyperactivity, bruxism, and hand biting.

The other affected sibling (IV:3) of IV:2, a 9-year old girl, was born at full term weighing 2.8 kg (-0.8 SD) and head circumference reported within normal limits. She was delayed in all aspects of development; she sat unsupported at approximately 17 months of age. She remained nonambulatory and nonverbal with profound intellectual disability. Growth parameters at the time of evaluation were: height at -5.9 SD, weight at -3.3 SD and OFC at -2.7 SD. She had hypotonia in infancy, and neurologic evaluation showed muscle weakness and atrophy. She had no history of seizures. Behaviorally, she had autism and demonstrated hand and facial stereotypies, laughing spells, anxiety and mood changes, agitation, sleep problems, hyperactivity, bruxism, and hand biting.

Family 3

Family 3 is a Persian family with two affected sisters born to consanguineous parents who are first cousins. The proband (IV:2) was 11 years old at time of the evaluation. She was born at full term weighing 2.6 kg (-1.0 SD) with a head circumference of 31 cm. She had early developmental delays and remained nonverbal and nonambulatory with severe intellectual disability. Growth parameters at time of evaluation were weight 22 kg (-3.2 SD), height 131 cm (-1.2 SD) and head circumference 53 cm (+0.3 SD). She had low set ears and nystagmus, as well as a chest deformity. Neurologically, she demonstrated hypotonia in infancy and, at evaluation, was noted to have absent DTRs, muscle weakness and atrophy. She has no history of seizures. Behaviorally, she demonstrated hand and facial stereotypies, spells of screaming and laughing, inappropriate fear, body rocking and irritability. Hyperactivity, bruxism and hand-biting were not reported. Brain MRI at age 4 months demonstrated mild brain atrophy. EMG was normal and CytoSNP array genotyping did not reveal any pathogenic CNVs. Plasma amino acids were non-diagnostic.

The second affected sister (IV:1), age 15 years at evaluation, was born at full term weighing 2.43 kg (-1.4 SD) with a head circumference of 33 cm and had early developmental delay, first sat unsupported at 4 years of age, and remained nonverbal and nonambulatory with severe intellectual disability. Growth parameters at time of evaluation were weight 27 kg (-5.9 SD), height 153 cm (-1.4 SD) and head circumference 56 cm (+1.6 SD). She had low set ears and esotropia, as well as a chest deformity. Hyperactivity, bruxism and hand-biting were not reported. Neurologically, she demonstrated hypotonia in infancy and, at evaluation, had absent DTRs, muscle weakness and atrophy. There is no history of seizures. EMG was normal and CytoSNP array genotyping did not reveal any pathogenic CNVs. Plasma amino acids were non-diagnostic. Brain MRI at age 2 years was normal.

Family 4

Family 4 is a Mexican family with two affected siblings. There is possible unconfirmed distant consanguinity in the family. The female proband (II:1), 11 years old at the time of evaluation, was born at full term with no complications and weighed 3.6 kg (+0.4 SD). She spent 4 days in the hospital following birth due to feeding difficulties, which quickly resolved. She was delayed in all of her milestones; she rolled over and sat up but never crawled, and remains nonverbal and wheelchair-bound with severe intellectual disability. At 8 years, 6 months, height was -1.4 SD. Growth parameters at evaluation were: weight at -2.8 SD and head circumference at -2.1 SD. Neurologically, she demonstrated hypotonia in infancy, as well as hyporeflexia, dystonia, spasticity, muscle weakness and atrophy at evaluation. Her seizures first occurred at age 3 months and she continued to have poorly controlled myoclonic epilepsy at the time of evaluation despite treatment with valproic acid and Keppra. Medical history includes bilateral Achilles tendon release. Behaviorally, she demonstrated atypical hand movements, drooling, hyperactivity and self-injurious behavior (biting her own hands). EEG report demonstrated "frequent pseudo-periodic bursts of slowing which often contain either generalized or multifocal

sharp wave and spike.” Noncontributory genetic testing included karyotype, Fragile X and *MECP2* sequencing. A chromosome microarray identified a variant of unknown significance (arr[GRCh37] 11p13(31772852_32722172)x2~3) with inadequate evidence for pathogenicity.

The affected brother (II:2), had developmental delays and was diagnosed with Dravet Syndrome and myocolonic seizures in Mexico in early childhood. At 18 years, 5 months, height was -2.5 SD and weight was -3.4 SD. Neurologically, he demonstrated hypotonia in infancy and, although reportedly able to walk at one time, he subsequently became nonambulatory with muscle weakness and atrophy. He was described to have both staring episodes and generalized seizures. Behaviorally, he demonstrated drooling, laughing and screaming spells, self-injury and hyperactivity. EEG noted generalized background slowing and bilateral frontal spikes.

Family 5

Family 5 is a Turkish family with four affected individuals. The proband IV:4 (BAB9717) is a 9-year-old female who was referred to us at age 7 due to severe developmental delay. She was born to a 26-year-old G1P1 mother at term via normal spontaneous vaginal delivery after an uncomplicated pregnancy and delivery. Her birth weight was low at ~2300 g (-2.1 SD). She was hypotonic at birth and showed delays throughout early childhood for developmental parameters. She achieved head control at 8 months, however she never achieved the milestones of rolling over or sitting unsupported. She did not say any meaningful words. She developed mild spasticity and dystonia during her toddler years followed by severe hypotonia. She had insomnia and poor weight gain since early childhood. Her anthropometric measurements at age of 9 years revealed - weight: 13.5 kg (-3.89 SD), height: 105 cm (-4.67 SD) and OFC: 49.5 cm (-2SD). Physical examination showed mild dysmorphia with hypotelorism and tapering fingers, autistic features including hand and facial stereotypies, teeth grinding, anxiety and inappropriate laughing/screaming spells. Neurological evaluation showed severe hypotonia, decreased deep

tendon reflexes, muscle wasting and weakness. Cranial nerve exam was unremarkable except strabismus. Diagnostic laboratory work up including ammonia, lactate, plasma amino acid, urine organic acid, EMG/NCS were unremarkable. Brain MRI at age of 2 years 9 months showed slightly enlarged ventricles, mild cerebral and cerebellar volume loss (cerebrum more affected than cerebellum), slight paucity of deep white matter, and borderline low to normal size of corpus callosum.

IV:5 (BAB9718) is a 5-year-old male, brother to IV:4. Pregnancy was uncomplicated and he was born full term via normal spontaneous vaginal delivery. His birth weight was within normal limits, ~3400 g (40th percentile). Similar to his sister, he was severely hypotonic, had failure to thrive and never achieved speech or the motor milestone of rolling over. He had insomnia as well. Growth parameters at 5 years old were as follows: weight 11 kg (-3.86 SD), height 92 cm (-3.61 SD) and normal head circumference (50.5 cm, 36th percentile). He had mild facial dysmorphic features including hypotelorism, short philtrum, and strabismus. He was drooling profusely. He endorsed autistic features similar to his sister with hand and facial stereotypies, teeth grinding without anxiety, and inappropriate laughing/screaming spells. Neurological examination revealed severe hypotonia, decreased tendon reflexes, muscle weakness, and atrophy. Metabolic and EMG/NCS were unremarkable. Brain MRI was obtained at 9 months old and showed delayed myelination with no myelination in the frontal white matter, thin CC, mild cerebral and cerebellar atrophy, especially in the frontal and Sylvian fissure areas (cerebrum more affected than cerebellum).

IV:1 (BAB12000) is an 11-year-old male, paternal cousin to IV:4 and IV:5. He was born at term with a normal birth weight of ~3250 g (31st percentile). Pregnancy and delivery were uncomplicated. Similar to his cousins, he was also severely hypotonic in infancy. Although he showed delays similar to other affected individuals in his family, he gained the highest developmental skills; he was able to sit unsupported at 7 years old and he was able to say 3 meaningful words. However, he showed regression and lost these abilities at his last evaluation

at 11 years old. He had autistic features similar to affected cousins including hand stereotypies, inappropriate screaming and laughing spells. He also had insomnia since early childhood. Metabolic and EMG/NCS were unremarkable.

IV:2 (BAB12003) is a 15-month-old male, brother to IV:1. He was born full term with a birth weight of ~4000 g (81st percentile) after an uncomplicated pregnancy and delivery. He had insomnia and was severely hypotonic at birth. He then developed mild spasticity similar to his affected brother and cousins. Measurements at 15 months of age revealed: weight 9.4 kg (6th percentile), height 79 cm (54th percentile) and head circumference (46th percentile). He did not achieve any developmental skills including head control, unsupported sit, or single words. He showed decreased reflexes. He did not have dysmorphic features other than hypotelorism. Diagnostic laboratory work up including metabolic and EMG/NCS were unremarkable.

Family 6

Family 6 is an Egyptian family with two affected brothers born to consanguineous parents who are first cousins. The proband (IV:1), 11 years old at the time of evaluation, was born at full term and delayed in early developmental milestones. He first sat unsupported at 12 months of age and first walked at 36 months of age. His first words were also at 36 months but he progressed to have only a few words in his vocabulary. He was ultimately diagnosed with profound intellectual disability with an IQ of 12 and noted to have features concerning for autism with the Childhood Autism Rating Scale (CARS). Growth parameters at evaluation were: height at -0.2 SD, weight at -2 SD, and head circumference at -0.2 SD. Behaviorally, he demonstrated bruxism, laughing and screaming spells, body rocking, mood changes, hyperactivity, and occasional agitation or irritability. He had anxiety that improved after age 3 years. He had frontal bossing, deep set eyes, anteverted nares and down-turned corners of the mouth. Neurologically, he demonstrated hyperreflexia and spasticity, but had intact cranial nerves. EEG

was abnormal and notable for cortical and subcortical epileptogenic activity and he had a possible history of complex partial seizures (screaming followed by staring or unresponsiveness) that improved in frequency from once every 20 days to once every 60 days on carbamazepine. MRI done at 5 years and 4 months showed mild atrophic brain changes including subtle thinning of the anterior corpus callosum, mild prominence of the subarachnoid spaces and lateral ventricles anteriorly with megacisterna magna. Unremarkable laboratory evaluations included normal CBC, chemistry, serum amino acids and acyl carnitines, lactate, ammonia, CPK, and urine organic acids. He also had negative Fragile X repeat expansion testing.

The younger affected brother (IV:2), 8 years old at the time of evaluation, was born at full term and his early milestones were also delayed. He first sat unsupported at 9 months of age and first walked at 36 months of age. Like his brother, he had only a few words in his vocabulary at the time of evaluation. IQ testing revealed severe intellectual disability with an IQ of 28 and there was no concern for autism based on the CARS. Growth parameters at evaluation were: height at -0.8 SD, weight at -2 SD, and head circumference at -1.7 SD. Behaviorally, he demonstrated hyperactivity and aggression. His exam was notable for brachycephaly, frontal bossing, deep set eyes, anteverted nares and down-turned corners of the mouth. Neurologically, he also showed hyperreflexia and spasticity, and had intact cranial nerves. He had no history of seizures. MRI at 2 years 7 months noted subtle thinning of the anterior corpus callosum consistent with mild atrophic brain changes and incomplete maturation. Serum amino acids and acyl carnitines were normal.

Family 7

Family 7 is a Bangladeshi family with one affected individual. The proband is a 3-year-old girl that was born full term with no complications from two non-consanguineous parents. Her mother’s antenatal history was uneventful. At birth, weight was 2500 g (-2.1 SD) and occipital-

frontal circumference was 32 cm. She had neonatal jaundice on day 3 of life and was successfully treated with phototherapy. She was delayed in all of her milestones since early infancy; she attained head control and sitting at the age of 9 months, crawled at 24 months and started to walk at 30 months. Currently, she can walk short distance and stand with support. She has severe intellectual disability and does not follow simple commands. She never attained meaningful speech. At 30 months, weight was – 2 SD. Her eye contact was poor, and she showed abnormal behavior, including irritability and tendency to isolation. She also exhibited stereotyped movements of the hands and the head (nodding) and showed signs of psychomotor regression. On the neurological examination, generalized hypotonia and mildly increased deep tendon reflexes in the lower limbs were evident. Muscle power and trophism were mildly reduced. On her last clinical evaluation at the age of 3 years her height was 95 cm (0.7 SD) and weight 12.8 Kg (-0.3 SD). There are no concerns with her vision or hearing. Since the age of 10 months she has had seizures which have included generalized tonic-clonic or myoclonic seizures (mainly involving the lower limbs), usually associated with loss of consciousness and upward eye deviation. Seizure episodes have variable duration (ranging between minutes to hours) and occurred up to 20-25 times at onset. Several anti-epileptic medications have been trialed including valproic acid, levetiracetam and clobazam, with only partial clinical response. EEG revealed generalized background slowing and bilateral frontal spikes; high amplitude delta waves are also noted during ictal period. Brain MRI showed a simplified gyral pattern and posteriorly slender corpus callosum (not shown). Whole exome sequencing of the girl was performed and identified a homozygous missense mutation in NTNG2 (NM_032536.2: c.599C>T; p.Ser200Leu). The variant was confirmed by Sanger.

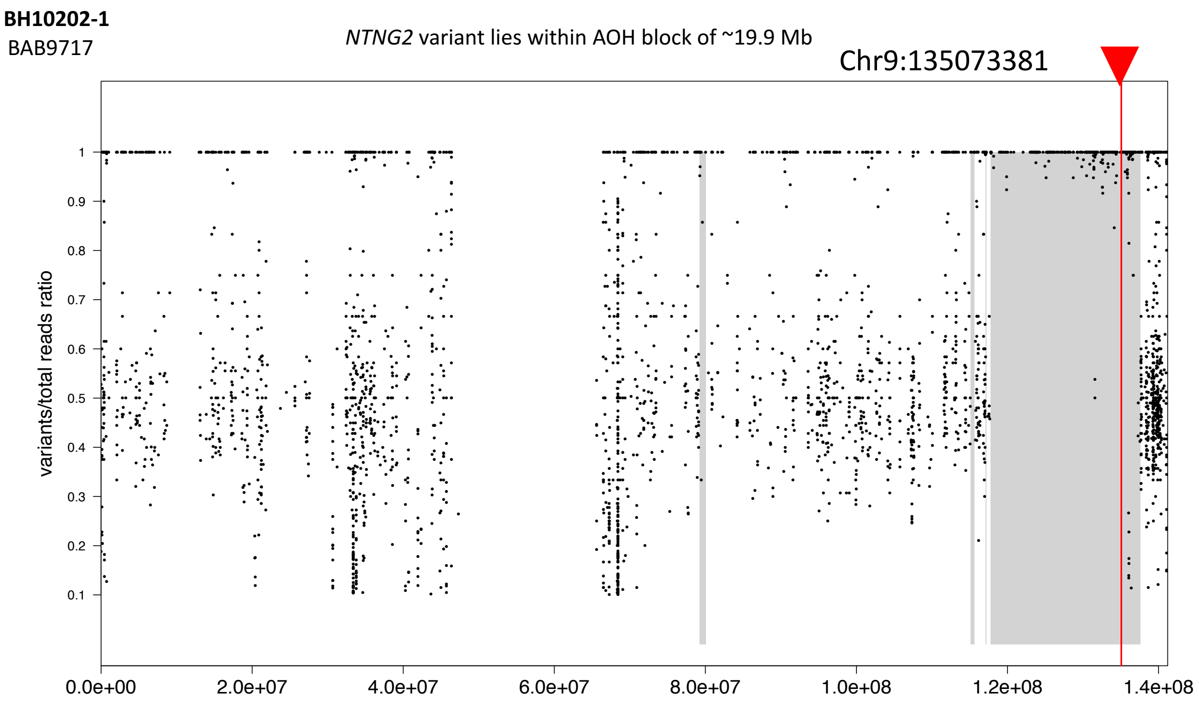

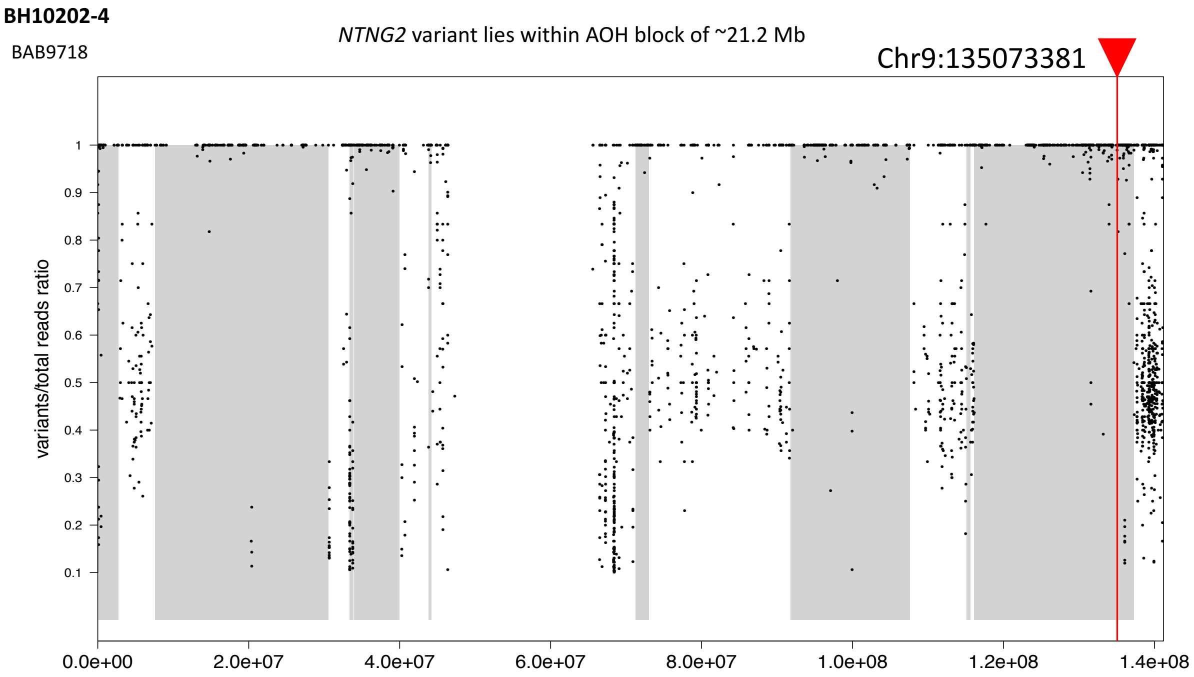

Figure S1: AOH plots for Family 5 based on B-allele frequencies. Top: IV:4:*NTNG2* variant lies within an AOH region of ~19.9 Mb. Bottom: IV:5:*NTNG2* variant lies within AOH region of ~21.2 Mb.

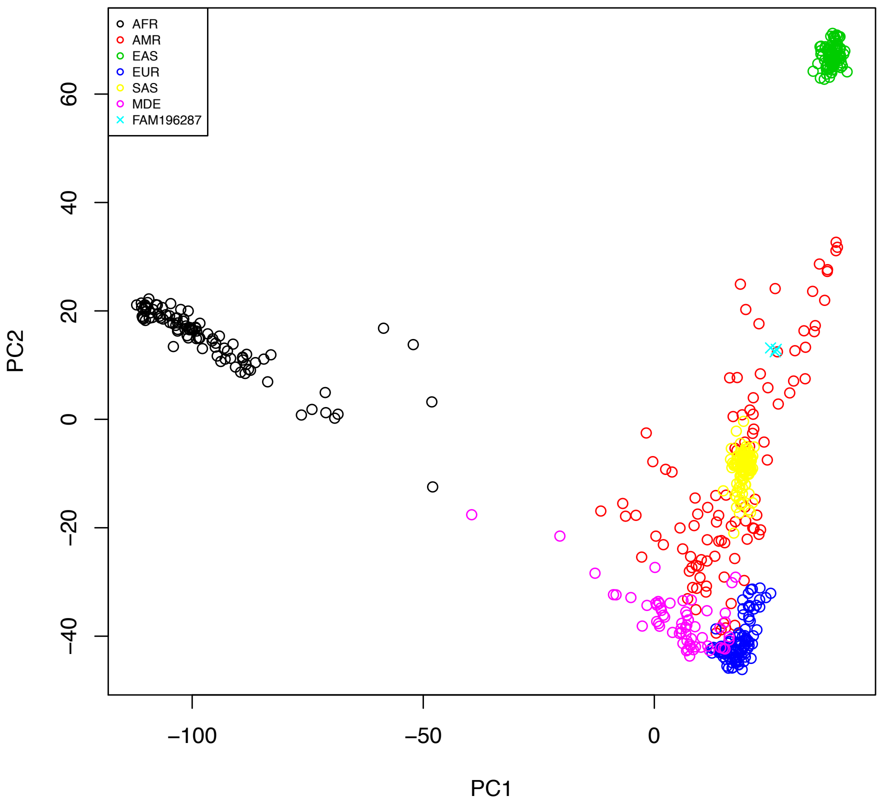

Figure S2: Representative PCA plot of Family 4 demonstrating close relatedness.

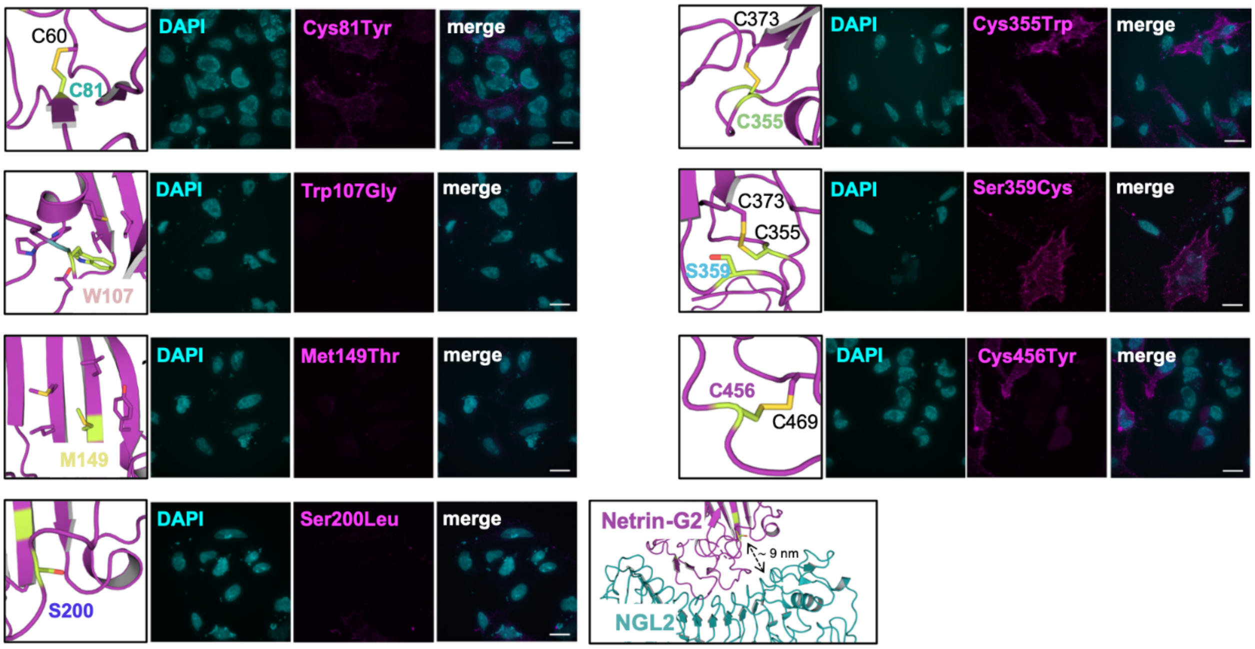

Figure S3: Structural views and cell surface expression.

Zoomed structural views of the netrin-G2 residues mutated in the variants presented here are shown next to representative images from the cell-surface expression assay and next to a zoomed structural view of the netrin-G2 binding interface to NGL2 for Ser200. For the cell-surface expression assay, Flag-tagged protein was detected by cell-surface immunostaining (magenta). DAPI (blue) highlights cell nuclei. Scale bar is 15 µm.

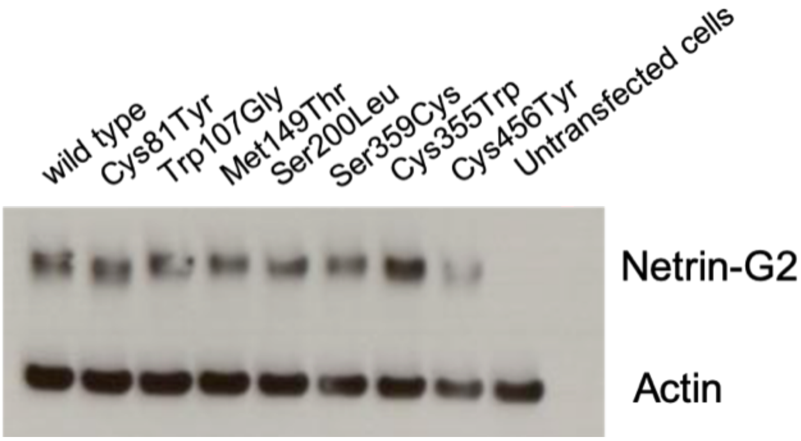

Figure S4: Western blot validation of cell-surface assay

Lysates from HeLa cells transfected with Flag-tagged NetrinG2 were probed with anti-Flag (top band) and anti-actin (lower band), as indicated.

Table S1: Clinical characteristics of individuals with homozygous *NTNG2* variants.

See associated excel file. Abbreviations: GA, gestational age at birth; y, years; m, months; FTT, failure to thrive; CC, corpus collosum; WM, white matter; VPA, valproic acid; NA, not ascertained/unknown; SD, standard deviation (when not directly provided back-calculated from CDC growth charts), WNL, within normal limits

Table S2	Family 1	Family 2	Family 3	Family 4	Family 5	Family 6	Family 7
***NTNG2* variants (hg19, NM_032536.3)**	g.135117272G>A, c.1367G>A, p.(Cys456Tyr)	g.135073458T>G, c.319T>G, p.(Trp107Gly)	g.135114512C>G, c.1076C>G, p.(Ser359Cys)	g.135114501C>G, c.1065C>G, p.(Cys355Trp)	g.135073381G>A, c.242G>A, p.(Cys81Tyr)	g.135073585T>C, c.446T>C, p.(Met149Thr)	g.135073738C>T, c.599C>T, p.(Ser200Leu)
**ROH/ size**	chr9:127,177,736-139,562,993 11.22 Mb	chr9:124,632,735 -136,404,775 11.77Mb	chr9:131,788, 926-141,044,489 9.25 Mb	chr9:134,386,759-135,763,816 1.4 Mb	IV:4 chr9:117, 792,583-137,630,587, 19.9 Mb IV:5 chr9:116,152,441-137,293,761 21.2 Mb	minimum shared ROH chr9:128,070,227-140,218,325, ~ 12Mb	chr9:131,261,128-132,241,791 ~1 Mb
**FATHMM**	Damaging (-6.65)	Damaging (-2.17)	Tolerated (0)	Damaging (-3.4)	Damaging (-2.09)	Tolerated (-0.92)	Damaging, (-1.73)
**Mutation Assessor**	High (4.725)	High (3.785)	Medium (3.5)	High (4.42)	High (3.745)	Medium (2.61)	Medium (2.96)
**MutationTaster**	Disease causing (1)	Disease causing (1)	Disease causing (1)	Disease causing (1)	Disease causing (1)	Disease causing (1)	Disease causing (1)
**PolyPhen-2**	Probably damaging (0.999)	Probably damaging (1.00)	Probably damaging (1.00)	Probably damaging (1.00)	Probably damaging (1.00)	Probably damaging (0.997)	Probably damaging (1.00)
**SIFT**	Tolerated (0.06)	Deleterious (0.00)	Deleterious (0.02)	Deleterious (0.00)	Deleterious (0.00)	Deleterious (0.01)	Deleterious (0.00)
**PROVEAN**	Deleterious (-6.98)	Deleterious (-11.77)	Deleterious (-2.91)	Deleterious (-8)	Deleterious (-9.14)	Deleterious (-4.75)	Deleterious (-4.59)
**CADD**	25.8	28.6	28.12	27.3	27.1	26.1	32
**GERP++**	3.39	5.13	4.94	3.82	5	5.22	5.22
**Ensembl**	Not present	Not present	Not present	Not present	Not present	Not present	Not present
**EVS**	Not present	Not present	Not present	Not present	Not present	Not present	Not present
**GME**	Not present	Not present	Not present	Not present	Not present	Not present	Not present
**gnomAD**	Not present	Not present	Not present	Not present	Not present	Not present	Not present
**Iranome**	Not present	Not present	Not present	Not present	Not present	Not present	Not present
**In-house databases**	Not present (10,000)	Not present (10,000)	Not present (7,000)	Not present (60,039)	Not present (1,053	Not present (~10,000)	Not present (10,000)

					Turkish, ~13000 all)		

Table S2: Molecular details of all *NTNG2* variants including pathogenicity prediction and presence in genomic databases. GERP score calculated using UCSC Genome browser's GERP++ track with the average value for the specific nucleotide genomic position presented. ROH: region of homozygosity. Number of total individuals in local databases indicated in parentheses.

Family Number	Gene Name	Genome Build	Transcript ID	Genomic Location	HGVS.c	HGVS.p	Absent in control databases	Majority of prediction tools predict damaging	Other Notes
1	ASB6	GRCh38	ENST00000277458.5	chr9:129638578	c.593A>G	p.(Glu198Gly)	Yes	Yes	
2	SFTPA1	GRCh37/hg19	NM_001093770.2	chr10: 81372106	c.256G>A	p.(Gly86Arg)	No	No	
3	PNPLA7	GRCh37/hg19	NM_001098537.1	chr9:140444616	c.30+4T>C	?	No	No	
3	PRMT2	GRCh37/hg19	NM_001242864.1	chr21: 48068538	c.489+7G>A	?	No	Yes	
5	SARDH	GRCh37/hg19	NM_007101.3	chr9:136535834	c.2367C>G	p.(Ser789Arg)	No	No	Conflicting interpretations of pathogenicity, AR variants linked to Sarcosinemia {MIM# 268900}, homozygous in Family 5: IV:4 and IV:5.
6	TENM2	GRCh37/hg19	NM_001122679	chr5:167625990	c.3033C>T	p.=	No	No	Common synonymous variant at poorly conserved nucleotide with no predicted slicing effect.
6	ADAMTS13	GRCh37/hg19	ENST00000371929	chr9:136314957	c.2915G>A	p.(Arg972Gln)	No	No	Alternate amino acid is present in other species, and previously reported associated phenotype (Thrombotic thrombocytopenic purpura) is inconsistent with presentation of affected individuals.
6	KLHDC4	GRCh37/hg19	NM_017566.4	chr16:87743027	c.1291C>G	p.(Pro431Ala)	No	No	Alternate amino acid is present in other species
6	ATRX	GRCh37/hg19	NM_000489	chrX:76889242	c.4810-42T>A	intronic variant	Yes	No	Deep intronic variant at poorly conserved nucleotide unlikely to effect splicing.

Table S3: List of alternate variants segregating with clinical phenotype and rationale for exclusion. Alternate candidates for Family 4 and 7 were not identified.

Supplemental Methods

Cell-surface expression assays

Wild type human (GenBank: BC013770.1) and variants netrin-G2 (residues Asp18-Arg530, including the C-terminal stop codon) were cloned into a pHLsec vector[1] with an N-terminal Flag tag following on from the vector secretion signal sequence. HeLa cells cultured on glass cover slips were transiently transfected with the plasmids described above and transfection success was assessed by Western blotting. Anti-Flag (Sigma-Aldrich, Cat No. F1804) was added to the cells at a concentration of 5 µg/ml. Cells were incubated for 1 hour on ice and washed with phosphate buffer saline (PBS). Cells were fixed in 4% paraformaldehyde (20 min, on ice), immunostained with Cy3-labeled anti-mouse antibody at a concentration of 50 µg/ml (Abcam, Cat No. ab97035), counter-stained with DAPI and mounted. For one assay, four cover slips of each variant were prepared and the assay was done in triplicates. Three representative samples of cells were imaged from each coverslip and images were analyzed using Fiji/ImageJ. Briefly, for each sample, the DAPI image was used to create a binary mask, which was applied to the Cy3 image. After thresholding the resulting image, the total number of pixels (Cy3 area) was measured and divided by the total number of pixels from the DAPI image (DAPI area) to obtain the adjusted area. Measurements of adjusted area from each coverslips were averaged and counted as one value (n = 12). We performed one-way ANOVA with Tukey Test for statistical analysis.

Western blot detection

HeLa cell lysates were probed with mouse anti-Flag (Sigma-Aldrich, Cat No. F1804) and mouse anti-actin (Abcam, Cat No. ab3280) mAbs in combination with Horseradish peroxidase (HRP)-labelled anti mouse mAbs (Sigma-Aldrich, Cat No. A0168). The signal was captured on autoradiography films (Amersham Hyperfilm ECL).

siRNA Experimental Methods and Analysis:

Constructs and antibodies

Small interfering RNA (siRNA) targeting mouse *Ntng2* or scrambled siRNA (catalog no. s100887 and 4390846; Thermo Fisher Scientific, Waltham, MA). The antibodies used for immunostaining were anti-Map2 (Microtubule Associated Protein 2) and Goat anti-Guinea Pig Alexa Fluor 568 (Invitrogen, Carlsbad, CA).

Neuro 2a cell culture and transfection

Mouse Neuroblastoma N2a cells were obtained from American Type Culture Collection (Manassas, Virginia). The N2a cells were cultured in Dulbecco's modified Eagle's medium (DMEM, Gibco, Waltham, MA) supplemented with 10% Fetal Calf Serum (Merck, Darmstadt, Germany), 1% Penicillin-Streptomycin (Merck, Darmstadt, Germany), 1% MEM Non-essential Amino Acid Solution (Merck, Darmstadt, Germany) and 1% Alanyl-glutamine (Merck, Darmstadt, Germany). Cultures were maintained at 37 °C with atmospheric air and 5% CO_2 and passaged when 90% confluent.

Transfection

The transfection experiment was carried out with *NTNG2* siRNA or the corresponding scrambled siRNA (siRNA ctrl). To form siRNA-lipid complexes, 12 pmol siRNA was mixed with 2 µl Lipofectamine RNAiMAX (Invitrogen, Carlsbad, CA) reagent in Opti-MEM (Gibco, Waltham MA).

Reverse transcription – quantitative PCR (RT-qPCR)

RNA from the N2a cells was extracted using the NucleoSpin RNA kit from Macherey-Nagel after 30h post transfection. Following RNA isolation, cDNA was synthesized using the iScript cDNA Synthesis Kit (Bio-Rad, Veenendaal, Netherlands) and purified using the NucleoSpin Gel and PCR Clean-up kit (Macherey-Nagel, Bethlehem, PA). Real-time PCR was performed using

GoTaq qPCR Master Mix (Promega, Leiden, Netherlands). The thermal cycling program consisted of 95°C for 10 min, 40 cycles of 95°C for 15 sec and 60°C for 30 sec. The relative gene expression was calculated using the $2^{-\Delta\Delta CT}$ method. The mus musculus peptidylprolyl isomerase A (*mPpia*) gene was used to normalize variations in the amount of starting material. Primer sequences are available upon request.

Fluorescence immunocytochemistry

For immunocytochemistry, cells were grown on glass coverslips coated with Poly-L-Ornithine (PLO, Sigma-Aldrich, Saint Louis, MO) and transfected with siRNA as described above. 30h after transfection cells were fixed with paraformaldehyde (4% in PBS, Thermo Fisher Scientific, Waltham, MA) for 15 min at room temperature. The coverslips were sequentially incubated in 5% normal goat serum, the primary antibody (overnight, 4°C), a secondary antibody, and Hoechst 33342 (Invitrogen, Carlsbad, CA). Both primary and secondary antibodies were diluted in 1% normal goat serum. The coverslips were mounted on slides with Fluorescence Mounting Medium (DAKO, Carpinteria, CA) and images were captured on a fluorescence microscope (Zeiss Axio Imager Z1, Oberkochen, Germany) equipped with a camera and the imaging software Zeiss Zen 2 (Oberkochen, Germany).

Image Analysis

To determine the percentage of cells with neurites, total cell count and cells with neurites were quantified manually. For each group, cells were examined in 14 random non-overlapping images. Images were then semi-automatically traced using Neurolucida 360 software (MBF Bioscience, Wilmington, VT). From each picture the two cells with the longest neurite length were reconstructed. In total 16 cells were reconstructed. Exclusion criteria for defining a neurite: length <3.5 μm; thickness <0.05 μm. Traces were imported into Neurolucida Explorer 360 (MBF Bioscience, Wilmington, VT) and the following sets of data were collected: number of primary neurites, number of branch points, total neurite outgrowth length (μm), and convex hull area (μm^2). Convex Hull analysis is used to define dendritic field area.

Statistical Analysis

Statistical analysis of experiments comparing data from two groups were conducted using unpaired Student's t-tests if data met the normality, otherwise, MannWhitney U tests was conducted. Results represent mean ± SD and P values < 0.05 were considered statistically significant. *p < 0.05, **p <0.01, ***p < 0.001.

Sequencing methods

Family 1 p.(Cys456Tyr)

This research study was approved by the Medical Ethical Committee Arnhem-Nijmegen, The Netherlands and the Mashhad University of Medical Sciences- Iran Institutional Review Boards. Written informed consent was obtained. Genomic DNAs were extracted from whole blood by standard protocol and then high-quality of probands' DNAs were shipped for exome sequencing, using Illumina HiSeq 2500, Q30≥80% (Novogene, Beijing). The procedure of ES including exome capture by using Agilent SureSelect Human All Exon V6 Kit and 50× of sequencing depth resulting in sequences of greater than 100 bases from each end of the fragments. The GRCh37/UCSC hg19 was considered as a reference genome. VarScan version 2.2.5 and MuTec and GATK Somatic Indel Detector were used to detect SNV and InDels, respectively. The protocol to interpret potential pathogenic variants was described previously[2].

Family 2 p.(Trp107Gly)

This research study was approved by the University of California San Diego IRB, and the Shahid Sadoughi University of Medical Sciences in Yazd, Iran, Institutional Review Boards. Written informed consent was obtained. ES was performed on DNA extracted from the proband (VI-2) by Macrogen, South Korea, using a SureSelect V6-Post Capture Kit (Agilent Genomics) and HiSeq 4000 sequencer (Illumina). The high-quality data were filtered for coding (nonsynonymous,

splicing, or truncating) variants followed by the removal of common variants (minor allele frequency >0.001) reported in publicly available population databases and in-house exome sequencing data on 631 control subjects. Pathogenicity and potential functional effects of the selected variants were assessed using in silico mutation prediction tools (e.g., SIFT, PolyPhen-2, MutationTaster, and PredictSNP). Selected variants were reviewed for previously reported phenotypes and functional and expression data based on OMIM, Genecards, and PubMed databases. Subsequently, those variants predicted to be damaging were considered as promising candidates for validation and segregation analysis.

Family 3 p.(Ser359Cys)

This research study was approved by the UCLH and NHNN, and the Ahvaz Jundishapur University of Medical Sciences, Iran, Institutional Review Boards. Written informed consent was obtained. Blood DNA was extracted using Qiagen reagents (Qiagen Inc., USA), then subjected to exome capture with the Illumina Rapid Capture 37 Mb Enrichment kit. Sequencing with 150-bp paired-end reads was performed using Illumina's HiSeq4000 instrument (Illumina, Inc., USA), resulting in >94% recovery at 10× coverage and >85% recovery at 20x coverage. GATK best practices pipeline was used for SNP and INDEL variant identification (http://www.broadinstitute.org/gatk/). Variants were annotated with in-house software[3] and homozygous variant prioritization was done using custom Python scripts (available upon request) to keep variants with MAF <0.001 in our sequenced cohort, or with high scores for likelihood to damage protein function. Sanger sequencing was used for segregation testing in all available family members.

Family 4 p.(Cys355Trp)

Using genomic DNA from the proband and parents, the exonic regions and flanking splice junctions of the genome were captured using the IDT xGen Exome Research Panel v1.0. Massively parallel (NextGen) sequencing was done on an Illumina system with 100bp or greater paired-end reads. Reads were aligned to human genome build GRCh37/UCSC hg19, and analyzed for sequence variants using a custom-developed analysis tool. Additional sequencing technology and variant interpretation protocol has been previously described[4]. The general assertion criteria for variant classification are publicly available on the GeneDx ClinVar submission page (http://www.ncbi.nlm.nih.gov/clinvar/submitters/26957/). Sanger sequencing was used for segregation testing in the parents and affected brother.

Through manual evaluation of the VCF file, a shared haplotype was identified between the parents leading to a ROH in the proband from chr9:134386759-135763816 (hg19).

This ROH was approximately 1.4 Mb. However, there are only 29 informative SNPs across this region, so the exact region may be larger. Using the Rutgers tool (http://compgen.rutgers.edu/map_interpolator.shtml), this equates to 2.08 cM, implying a founder event ~48 generations ago. See representative PCA plot showing their close relatedness in Figure S2. Haplotype analysis and PCA plot generation was performed as per previously described [5,6].

Family 5 p.(Cys81Tyr)

All individuals and their family members reported here provided informed consent in accordance with the Baylor-Hopkins Center for Mendelian Genomics (BHCMG) research protocol (Baylor College of Medicine, IRB protocol number: H-29697). Exome sequencing (ES) was performed on the proband BAB9717 (Family 5, IV:4) and affected sibling BAB9718 (Family 5, IV:5) at the Human Genome Sequencing Center at Baylor College of Medicine using methods previously described[7]. Exome analyses for candidate variants was performed by comparing shared

variants in both affected siblings and filtering candidate variants with genomic data from public databases, including 1000 Genomes database (1000G, http://www.1000genomes.org), Exome Variant Server (EVS, http://evs.gs.washington.edu/EVS), the Atherosclerosis Risk in Communities Study Database (ARIC, http://drupal.cscc.unc.edu/aric/), genome Aggregation Database (gnomAD, http://gnomad.broadinstitute.org/), Exome Aggregation Consortium (ExAC, http://exac.broadinstitute.org/), and our in-house exome database (~13,000 individuals) at the Baylor College of Medicine Human Genome Sequencing Center. We further filtered variants based on pathogenicity prediction and conservation scores, with filtering out variants with low conservation scores (PhyloP, GERP), predicted to be tolerated/benign by at least 4 out of 5 pathogenicity prediction tools (FATHMM, MutationTaster, PolyPhen-2, SIFT, PROVEAN) and a CADD score under 14. ES analyses revealed a shared homozygous rare missense variant in *NTNG2* in both affected siblings that is predicted to be disease causing (Table 1). Analyses of absence of heterozygosity (AOH) regions from exome data using BafAOHCalculator demonstrated that both siblings IV:4 and IV:5 showed total AOH regions of ~312 and ~333 Mb respectively, consistent with reported parental consanguinity[8]. The rare *NTNG2* variant was present in an AOH region of ~19.9 Mb in IV:4 and ~21.2 Mb in IV:5 (Figure S3). Notably, these regions were not the largest AOH regions observed in either sibling (~26 Mb and ~31 Mb, respectively). The candidate *NTNG2* variant was confirmed to be homozygous in both affected siblings and heterozygous in unaffected parents using standard PCR amplification and Sanger sequencing techniques. Two affected paternal cousins with similar clinical features were identified; PCR amplification and Sanger sequencing for the missense *NTNG2* variant in this family co-segregated with the neurological phenotype in accordance with expectation for an autosomal recessive trait.

Family 6 p.(Met149Thr)

This study was approved by the institutional review boards of the Faculty of Medicine at Cairo University and Boston Children's Hospital. Subjects were identified and evaluated in a clinical setting, and biological samples were collected for research purposes after obtaining written informed consent.

Whole exome sequencing and data processing were performed by the Genomics Platform at the Broad Institute of Harvard and MIT (Broad Institute, Cambridge, MA, USA). We performed whole exome sequencing on DNA samples (>250 ng of DNA, at >2 ng/ul) using Illumina exome capture (38 Mb target). Our exome-sequencing pipeline included sample plating, library preparation (2-plexing of samples per hybridization), hybrid capture, sequencing (150 bp paired reads), sample identification QC check, and data storage. Our hybrid selection libraries cover >90% of targets at 20x and a mean target coverage of ~100x. The exome sequencing data was de-multiplexed and each sample's sequence data were aggregated into a single Picard BAM file.

Exome sequencing data was processed through a pipeline based on Picard, using base quality score recalibration and local realignment at known indels. We used the BWA aligner for mapping reads to the human genome build 37 (hg19). Single Nucleotide Polymorphism (SNPs) and insertions/deletions (indels) were jointly called across all samples using Genome Analysis Toolkit (GATK) HaplotypeCaller package version 3.4. Default filters were applied to SNP and indel calls using the GATK Variant Quality Score Recalibration (VQSR) approach. Lastly, the variants were annotated using Variant Effect Predictor (VEP). For additional information please refer to Supplementary Section 1 in ExAC paper[9]. The variant call set was uploaded on to seqr and analysis was performed using the various inheritance patterns. Sanger sequencing was used for segregation testing.

Family 7 p.(Ser200Leu)

This study was approved by local institutional IRB/ethical review boards of UCLH and NHNN, and written informed consent was obtained prior to genetic testing from the family involved. Clinical details were obtained through medical file review and clinical examination. Genomic DNA was extracted from peripheral blood samples according to standard procedures. WES using a trio approach was performed as described elsewhere[10], and our bioinformatics filtering strategy included screening for only exonic and donor/acceptor splicing variants. In accordance with the pedigree and phenotype, priority was given to rare variants (<0.01% in public databases, including 1,000 Genomes project, NHLBI Exome Variant Server, Complete Genomics 69, and Exome Aggregation Consortium [ExAC v0.2]) that were fitting a recessive (homozygous or compound heterozygous) or a *de novo* model and/or variants in genes previously linked to developmental delay, intellectual disability and other neurological disorders.

Funding

HH is supported by the Rosetree Trust, Ataxia UK, MSA Trust, Brain Research UK, Muscular Dystrophy UK, Muscular Dystrophy Association (MDA USA), Higher Education Commission of Pakistan, The MRC (MR/S01165X/1, MR/S005021/1, G0601943), Wellcome Trust (WT093205MA, WT104033AIA and the Synaptopathies Strategic Award, 165908) and the National Institute for Health Research University College London Hospitals Biomedical Research Centre.

HvB was supported by the European Union's Seventh Framework Program (Gencodys; grant 241995). DLP is recipient of a CAPES Fellowship, Brazil (99999.013311/2013-01).

Exome sequencing and interpretation work was supported by NIH grants R01NS048453, R01NS052455 and Howard Hughes Medical Institute (to JGG). Genome sequencing was performed through an in-kind donation of sequencing from Human Longevity Inc, and from the Rady Children's Institute of Genomic Medicine. We acknowledge the Broad Institute (U54HG003067 to E. Lander and UM1HG008900 to D. MacArthur and H. Rehm) and the Yale

Center for Mendelian Disorders (U54HG006504 to R. Lifton and M. Gunel) for access to exome sequencing.
Work for Family 5 was supported in part by the National Institutes of Health, National Institute of Neurologic Disorders and Stroke [R35 NS105078], a jointly funded National Human Genome Research Institute (NHGRI) and National Heart, Lung, and Blood Institute (NHLBI) grant to the Baylor-Hopkins Center for Mendelian Genomics [UM1 HG006542], and the Muscular Dystrophy Association, grant #512848 awarded to JRL. TM is supported by the Uehara Memorial Foundation. JEP is supported by NHGRI [K08 HG008986]. DP is supported by the Clinical Research Training Scholarship in Neuromuscular Disease partnered by American Brain Foundation (ABF) and Muscle Study Group (MSG), and NIH - Brain Disorders and Development Training Grant (T32 NS043124-17)
Sequencing and analysis for Family 6 were provided by the Broad Institute of MIT and Harvard Center for Mendelian Genomics (Broad CMG) and was funded by the National Human Genome Research Institute, the National Eye Institute, and the National Heart, Lung and Blood Institute grant UM1 HG008900 to Daniel MacArthur and Heidi Rehm. CAW is supported by NINDS grant R01NS035129, and is an Investigator of the Howard Hughes Medical Institute. CMD is supported by the NIH/NIMH Translational Post-Doctoral Training in Neurodevelopment T32 MH112510.
ES in funded by a Wellcome Trust grant (grant number 202827/Z/16/Z) and CZ is the recipient of a Wellcome Trust DPhil studentship.
Family 2 and Family 7 were collected as part of the SYNaPS Study Group collaboration funded by The Wellcome Trust and strategic award (Synaptopathies) funding (WT093205 MA and WT104033AIA). This research was conducted as part of the Queen Square Genomics group at University College London, supported by the National Institute for Health Research University College London Hospitals Biomedical Research Centre.

Acknowledgements

We thank Omneya G. Afify, Azza A. Oraby and Ola O. Shaheen for project supervision at Cairo University.

We thank Richard M. Parton and the MICRON imaging facility (http://micronoxford.com, supported by Wellcome Strategic Awards 091911/B/10/Z and 107457/Z/15/Z) for technical advice and access to equipment.

We thank Eunjoon Kim and Shigeyoshi Itohara for helpful review and comments on the manuscript.

We thank Lariza Rento, Robert Sean Hill, and Laura Isacco for technical assistance with Family 6.

Author Contributions

Conceptualization, R.M.; H.V.B.; Supervision H.H., J.R.L., G.S., H.G., C.A.W., J.G.G., E.S., H.V.B., R.M.; Formal Analysis, C.Z., V.O., L.S., E.S.; Investigation-Family 1: A.R., D.L.P., E.V.B., J.R.V.; Family 2: N.M., H.G., G.S., R.A.M., A.S.; Family 3: V.S., Y.V.M., M.D., A.S.; Family 4: J.K., A.P., L.D.; Family 5: J.P., A.G., T.M., D.P., Z.C., E.K., S.J., J.E.P., J.R.L.; Family 6: C.M.D., J.N.P., N.M.A., L.A.S., E.Y., C.A.W.; Family 7: S.E., V.S., S.B. H.H.; Writing- Original Draft, C.M.D, J.P., C.Z., R.M., E.S., Writing-Revision, R.M., H.V.B, J.R.L., J.N.P., C.A.W., E.S.

Supplemental References

[1] Aricescu A.R., Lu W., and Jones E.Y. (2006). A time- and cost-efficient system for high-level protein production in mammalian cells. Acta Crystallogr. D Biol. Crystallog. *62*, 1243-1250.

[2] Rad, A., Altunoglu, U., Miller, R., Maroofian, R., James, K.N., Caglayan, A.O., Najafi, M., Stanley, V., Boustany, R.M., Yesil, G., et al. (2019). MAB21L1 loss of function causes a syndromic neurodevelopmental disorder with distinctive cerebellar, ocular, craniofacial and genital features (COFG syndrome). J. Med. Genet. *56*, 332-339.

[3] Dixon-Salazar, T.J., Silhavy J.L., Udpa N., Schroth J., Bielas S., Schaffer A.E., Olvera J., Bafna V., Zaki M.S., Abdel-Salam G.H., et al. (2012). Exome sequencing can improve diagnosis and alter patient management. Sci. Transl. Med. *4*, 138ra78.

[4] Retterer K., Juusola J., Cho M.T., Vitazka P., Millan F., Gibellini F., Vertino-Bell A., Smaoui N., Neidich J., Monaghan K.G., et al. (2016). Clinical application of whole-exome sequencing across clinical indications. Genet. Med. *18*, 696-704.

[5] Burns, D.T., Donkervoort, S., Muller, J.S., et al. (2018). Variants in EXOSC9 Disrupt the RNA Exosome and Result in Cerebellar Atrophy with Spinal Motor Neuronopathy. Am. J. Hum. Genet. *102,* 858-873.

[6] Ying D., Sham P.C., Smith D.K., Zhang, L., Lau Y.L., Yang W. (2015). HaploShare: identification of extended haplotypes shared by cases and evaluation against controls. Genome Biol. *16,* 92.

[7] White, J.J., Mazzeu, J.F., Coban-Akdemir, Z., Bayram, Y., Bahram- beigi, V., Hoischen, A., van Bon, B.W.M., Gezdirici, A., Gulec, E.Y., Ramond, F., et al. (2018). WNT signaling perturbations underlie the genetic heterogeneity of Robinow syndrome. Am. J. Hum. Genet. *102*, 27–43.

[8] Karaca E., Posey J.E., Coban-Akdemir Z., Pehlivan D., Harel T., Jhangiani S.N., Bayram Y., Song X., Bahyrambeigi V., Yuregir O.O. et al. (2018). Phenotypic expansion illuminates multilocus pathogenic variation. Genet Med. *20*, 1528-1537.

[9] Lek M., Karczewski K.J., Minikel E.V., Samocha K.E., Banks E., Fennell T., O'Donnell-Luria A.H., Ware J.S. et al. (2016). Exome Aggregation Consortium. Analysis of protein-coding genetic variation in 60,706 humans. Nature. *18*, 285-91.

[10] Mencacci N.E., Kamsteeg E.J., Nakashima K., R'Bibo L., Lynch D.S., Balint B., Willemsen M.A., Adams M.E., Wiethoff S., Suzuki K.et al. (2016). De Novo Mutations in PDE10A Cause Childhood-Onset Chorea with Bilateral Striatal Lesions. Am. J. Hum. Genet. *98*, 763-771.
